# Low-level visual and auditory features predict preferences of commercials

**DOI:** 10.3389/fpsyg.2026.1918605

**Published:** 2026-10-29

**Authors:** Yael Valentina Parada, Inbal Gur-Arie, Dino Levy

**Affiliations:** 1 Sagol School of Neuroscience, Tel Aviv University, Tel Aviv, Israel; 2 School of Psychological Sciences, Tel Aviv University, Tel Aviv, Israel; 3 Coller School of Management, Tel Aviv University, Tel Aviv, Israel

**Keywords:** auditory-features, prediction, preference, video-commercials, visual-features

## Abstract

Video content has become a dominant form of digital marketing, increasing the need to understand which features make videos more appealing and effective. Prior studies showed that low-level visual features in static images affect saliency and consumer preferences, while low-level auditory features influence preferences for music clips. However, it remains unclear how these features and their interaction shape preferences for dynamic stimuli such as video commercials. In this study, 165 participants watched 100 video commercials and rated how much they liked each commercial and their willingness to pay (WTP) for the advertised product. We extracted low-level visual and auditory features from the commercials (e.g., colorfulness, scene frequency, zero-crossing rate) and used them as inputs to linear and non-linear prediction models. Both visual and auditory low-level features significantly predicted Liking and WTP, with the combined model performing best. Importantly, these low-level features also predicted the aggregate engagement metrics at the population level as measured on YouTube, demonstrating the generalizability of our findings and their external validity. These findings show that low-level sensory information influences consumer preferences and ad effectiveness and demonstrate how computational tools can be used to evaluate video effectiveness.

## 1 Introduction

With the growing use of video in marketing, education, and other domains, understanding which features make a video more likable and, consequently, more successful has become increasingly important. In particular, the rise of platforms like TikTok, YouTube Shorts, and Instagram Reels has made short-form video advertising a dominant and highly successful digital format. However, research suggests that this format is actively reshaping consumer behavior, shrinking attention spans and creating an oversaturated market that increases the difficulty for advertisements to stand out (Kumar, 2025). Consequently, marketers must carefully design visual and auditory elements to capture viewer attention within the first few seconds (Pieters et al., 2010; Yang Q. et al., 2025).

One approach to understanding these responses is to examine low-level sensory features, that is, basic perceptual properties of visual and auditory stimuli. From an information-processing perspective, sensory systems have limited capacity and therefore must efficiently select and represent incoming information (Shannon, 1948). Consistent with this view, early perceptual processing is thought to reduce redundancy and prioritize informative aspects of sensory input (Attneave, 1954; Barlow, 1961). In vision, for example, information is concentrated around features such as edges and corners rather than being uniformly distributed across an image (Attneave, 1954). Perception is also an active process in which attention selectively samples the environment (Gibson, 1979), suggesting that salient low-level cues can influence which aspects of a stimulus are prioritized. Accordingly, low-level visual and auditory features may provide important cues for understanding early attention allocation and subsequent affective responses to commercial content (Barnes, 2024; Zhang et al., 2025).

Neurophysiological evidence is consistent with this possibility. Early ERP components such as the N100 and P200 have been associated with attentional allocation and emotional responses to visual and auditory information (Van Paasschen et al., 2014; Shi et al., 2021). Eye-tracking and neuroimaging studies confirm that these sensory cues guide fixations, activate emotional and attentional networks, and influence recall in advertising contexts (Itti and Koch, 2000; Simmonds et al., 2020; Simola et al., 2020; Tavakoli et al., 2020).

These early perceptual processes are relevant to consumer evaluation, but advertising effectiveness extends beyond observable behavioral engagement. In advertising research, effectiveness is commonly conceptualized along three primary dimensions: Recall (how well the audience remembers the ad), Attitude (the consumer’s feelings toward the product or brand), and Action (the specific behaviors taken after exposure) (Mindak, 1956). Existing research has predominantly relied on the behavioral dimension as a proxy for users’ level of engagement, primarily capturing observable interactions such as likes and shares (Trunfio and Rossi, 2021) overlooking cognitive and emotional dimensions of social media engagement (Hollebeek et al., 2014). Behavioral metrics therefore provide only an indirect indication of consumers’ internal evaluations. The present study focuses on the attitudinal dimension by examining advertisement likability and willingness to pay (WTP). Likability captures consumers’ affective evaluation of an advertisement, whereas WTP provides a monetary expression of subjective product value (Becker et al., 1964; Kalish and Nelson, 1991; Wertenbroch and Skiera, 2002). Examining both outcomes therefore makes it possible to assess whether low-level auditory and visual features are associated not only with affective preference but also with consumers’ economic valuation of advertised products.

Prior research has demonstrated that both visual and auditory properties of marketing stimuli influence attention, affective responses, and consumer behaviors. In short-form video specifically, visual characteristics such as pixel-level complexity and auditory features such as average spectral centroid are systematically related to engagement metrics including likes, shares, and comments (Xiao et al., 2024). Low-level features are particularly relevant to esthetic preference and likability because they can shape viewers’ emotional responses during early stages of processing (Coutinho and Cangelosi, 2011; Van Paasschen et al., 2014; Fong et al., 2025). However, most previous research examined either visual or auditory features in isolation, despite evidence that perception and valuation strongly depend on integrating multiple sensory modalities (Opoku-Baah et al., 2021; Williams et al., 2022).

Low-level visual features are derived from the statistical and visual properties of each image, encompassing basic spatial properties such as entropy and edges, as well as color properties such as hue, saturation, and luminosity (Berman et al., 2014; Iigaya et al., 2021). These features form the foundation of early visual perception, influencing how consumers evaluate products and advertisements (Pieters et al., 2010; Krishna, 2012; Sample et al., 2020; Kanuri et al., 2024). The strategic use of luminance, shape, and surface color can enhance product appeal and shape preferences (Sample et al., 2020). Even subtle variations in luminosity, hue, or chroma can shift brand perceptions and purchase intent (Labrecque and Milne, 2012; Jalali and Papatla, 2016; Dzyabura and Peres, 2024). Moreover, it has been shown that brightness increases arousal (Areni and Kim, 1994), that saturation is associated with perceived product size and stronger consumer responses (Hagtvedt and Adam Brasel, 2017), that colors such as green and red alter perceptions of healthiness and WTP (Kunz et al., 2020) and that overall colorfulness predicts valence and arousal (Pazda et al., 2024). Evidence on visual complexity is more mixed. Greater feature complexity can reduce brand attention (Kanuri et al., 2024), while other works show that higher color and feature complexity captures attention and increases engagement (Pieters et al., 2010; Shin et al., 2020). This is likely due to a non-linear relationship with an inverted U-shaped pattern between feature complexity and consumer liking (Overgoor et al., 2022).

Beyond shaping perceptions, low-level visual features have also been shown to predict subjective preferences across a variety of stimuli, including art, photographs, fractal art, faces, property listings, natural vs. man-made scenes, and consumer products (Kardan et al., 2015; Oren et al., 2020; Iigaya et al., 2021; Fu et al., 2024). These effects are partly driven by visual attention, where visually salient features capture gaze and increase selection probability, where greater attention is associated with faster and more frequent choices (Pieters et al., 2010; Milosavljevic et al., 2012; Towal et al., 2013). Computational models of visual attention, including saliency models and the attention drift-diffusion model, similarly demonstrate how low-level visual information can influence attentional priority and subsequent choice and response times (Krajbich et al., 2010, 2012; Tavares et al., 2017).

Over the past decades, research in vision neuroscience has shown that saliency models estimate attentional priority by combining visual features (e.g., color, luminance, orientation) mimicking the brain’s bottom-up, stimulus-driven mechanism (Itti and Koch, 2001). These models reliably predict eye fixations, esthetic evaluations, and gaze allocation in real-world and marketing contexts (Foulsham and Underwood, 2008; Wong and Low, 2011; Hayes and Henderson, 2021; Lee et al., 2021). In dynamic stimuli, low-level visual properties such as brightness, contrast, flicker, and motion predict gaze allocation in video and advertising contexts (Le Meur et al., 2007; Mital et al., 2011; Breuer and Rumpf, 2015; Rumpf et al., 2020). Motion-related features play a dominant role, with motion-based saliency accounting for more fixations than contrast-based models (Mahapatra et al., 2008). Beyond attention, basic video properties (e.g., hue, saturation, brightness) and motion features also shape engagement (Zhou et al., 2021) and outperform high-level semantic descriptors in producing relevant recommendations and classifying movie genres (Deldjoo et al., 2015). Recent deep-learning approaches further link pixel-level visual engagement to consumer behavior (Yang J. et al., 2025). Taken together, these studies suggest that low-level visual features influence consumer responses not only by attracting attention but also by shaping evaluation and preference.

Visual information, however, is only one component of a commercial. Auditory information also influences emotional responses, product perceptions, and evaluations of marketing content (Yorkston and Menon, 2004; Coutinho and Cangelosi, 2011; Krishna, 2012; Corrigall and Schellenberg, 2013). For instance, lower pitch voices and music can lead consumers to infer larger product size (Lowe and Haws, 2017), while higher-tempo music has been associated with more positive affect toward travel and tourism ads (Barnes, 2024). Music frequency and timbre can also modulate perceived abstractness or concreteness of marketing messages (Sunaga, 2018).

Music perception research shows that manipulating timbre, amplitude, and fade-in influences perceived musicality (Gurariy et al., 2021) with effects that are often non-linear, since changing feature magnitude can either enhance or diminish preference depending on the stimulus’s baseline musicality (Gurariy et al., 2023). Acoustic features also predict emotional responses such as arousal and valence in computational models (Pons et al., 2016; Fong et al., 2025). Moreover, low-level auditory features have also been shown to predict preferences. Features such as tempo, timbre, and rhythm predict song popularity (Zangerle et al., 2019; Martin-Gutierrez et al., 2020). More broadly, properties such as tempo and speechiness contribute to the long-term success of musical albums (Boughanmi and Ansari, 2021). In advertising, spectral and rhythmic features predict commercial energy levels, with more energetic commercials viewed for longer (Yang et al., 2022) and an analysis of over 26,000 video ads showed that fast-tempo music increases purchase intentions for promotion-oriented video commercials (Lei et al., 2025).

Although it has been shown that low-level visual and auditory features independently influence consumer perception, most previous research has examined their effects in isolation despite their well-known interaction in shaping perception (Williams et al., 2022; Li et al., 2023). Visual information can refine auditory processing (Opoku-Baah et al., 2021), just as synchronous auditory signals facilitate visual perception (Vroomen and de Gelder, 2000). Congruent audiovisual cues guide attention more efficiently toward targets, whereas mismatched audio scatters gaze and slows reaction times (Chen et al., 2014; Einhäuser et al., 2024) and the auditory context can actively bias how participants interpret both ambiguous and unambiguous visual stimuli (Shams et al., 2000; Williams et al., 2022). In marketing, congruent combinations of visual and auditory elements, such as logos and associated sounds, can enhance consumer responses (Li et al., 2023). In multimedia contexts, while visual quality remains dominant, auditory degradation significantly alters overall user experience (Martinez et al., 2021).

Despite this evidence, relatively little research has examined whether combining low-level visual and auditory features improves the prediction of consumer preferences beyond either modality alone. Previous multimodal studies suggest that audiovisual interactions can influence consumer responses in both positive and negative ways (Liu et al., 2018), while combining information across modalities can improve predictive performance (Bougiatiotis and Giannakopoulos, 2018). However, these studies have largely relied on high-level semantic information, such as genres, event categories, or metadata, rather than fundamental low-level perceptual features.

The present study addresses this gap by analyzing low-level visual and auditory features extracted directly from video commercials, both separately and in combination, and relating them to consumer preferences. To our knowledge, few studies have directly compared unimodal and multimodal low-level features in predicting advertising preferences. We focus on basic properties such as luminosity and zero-crossing rate that reflect the raw signal of the video, to examine whether these features are associated with advertisement likability and consumers’ WTP, and whether combining both modalities improves predictive power compared with unimodal models. We further examine whether these sensory-preference relationships exhibit non-linear patterns. Methodologically, we extract multiple low-level visual and auditory features directly from video commercials and evaluate their predictive performance using out-of-sample cross-validation. Our research is guided by four questions: (1) How do low-level visual and auditory features individually relate to ad likability and WTP? (2) Does combining visual and auditory features improve the prediction of commercial preferences relative to single-modality models? (3) Which specific low-level features are the strongest predictors of ad likability and WTP? (4) Are these relationships linear, or do they exhibit non-linear patterns? We hypothesize that both visual and auditory features will significantly relate to ad likability and WTP, that multimodal models will provide greater predictive performance than unimodal models, and that some relationships will be non-linear. Together, these analyses provide insight into how low-level audiovisual features relate to consumer preferences and may inform the design of video advertising.

## 2 Methods

### 2.1 Participants

A total of 186 participants (19–63 years, mean = 26.23, SD = 6.18; 55.1% women) took part in the study, most being undergraduates at our university. The local ethics committee approved the study and written informed consent. The recruitment period ran from November 10, 2020, to November 17, 2024. To account for demographic variance, we conducted a linear mixed-effects regression to evaluate the effect of participants’ age on Liking and WTP (see Supplementary Appendix A2 for results).

### 2.2 Stimuli

We used 100 commercials extracted from YouTube, featuring 26 different products across 5 categories: beverages, breakfast items, dry goods, hygiene products, and snacks (the full list of products and links to the commercials is provided in Supplementary Appendix). Each product had 3–5 different commercials. The mean duration of the commercials was 6.19 s with a standard deviation of 0.42 s, aligning with the rapid rise of short-form advertising. YouTube was chosen as the source because it is the world’s second most popular social platform, and its short-form format is increasingly used to drive consumer engagement (Dong et al., 2023; Klempner and Epstein, 2023; Kumar, 2025). Furthermore, YouTube was selected because it provided access to the video content and relevant public metadata, including publication date, views, and likes. These data could be systematically collected using tools such as yt-dlp, allowing the same information to be obtained consistently across all commercials.

### 2.3 Procedure

Participants sat in front of a computer screen in the lab and watched a series of commercials for consumer products. The order of the commercials was randomized for each participant. After each commercial, two questions appeared one after the other (in a counterbalanced order). The questions were “How much are you willing to pay for this product?” where participants indicated their WTP for the product they just saw in the commercial by moving a slider on a scale between zero and one hundred tokens and “How much do you like the commercial?” where participants indicated their *Liking* for the commercial they just saw by moving a slider on a scale between zero and seven. Participants had a maximum of 15 s to indicate each of their ratings. Note that participants completed the task in a single session, which took around 25 min. The study design is presented in Figure 1.

**Figure 1:**
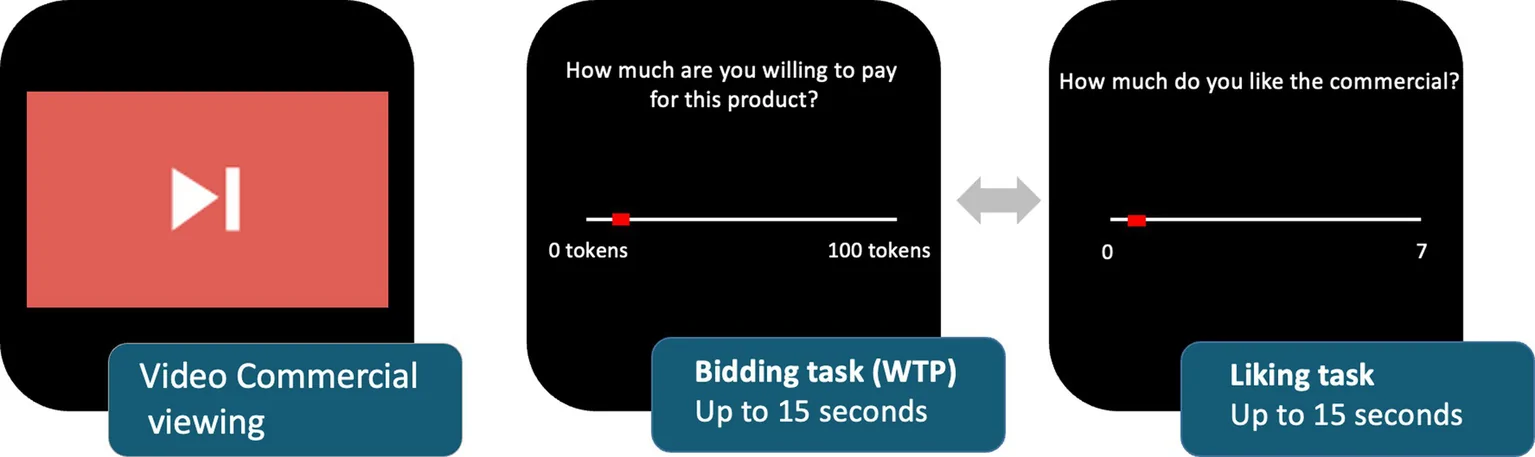
Study design. Participants viewed 100 different video commercials. After each commercial, they indicated their wtp for the advertised product and overall l*iking* for the commercial. These two questions were counterbalanced across trials.

### 2.4 BDM and willingness to pay

Methodologically, WTP has been extensively employed in contingent valuation frameworks to estimate changes in utility, particularly for non-tradable goods and services (Arrow et al., 1996; Choe et al., 1996; Ostensson, 2001). To ensure the experiment was incentive-compatible and that participants reported their subjective WTP for the products featured in the commercials, we used the standard BDM procedure (Becker et al., 1964), which has shown reliable and valid WTP estimates while reducing strategic response behavior. At the end of the study, the computer randomly selected one trial and generated a price between 0 and 100 tokens. If the participant’s bid exceeded the computer-generated random price, the participant buys the product at that price and keeps the remaining tokens. The 100 tokens were converted to ₪10, which corresponds to approximately $2.5. For instance, if the participant earned 48 tokens in the random trial, then they would get ₪4.8 plus their participation fee. Each participant received ₪80 as a participation fee. If the participant offered less than the random price, they did not win the product and received only the change left over from the participation fee. Tokens were used as an experimental currency to minimize immediate market-price anchoring (i.e., participants simply recalling and stating standard supermarket retail prices) while maintaining incentive compatibility through real monetary outcomes.

### 2.5 Analysis

#### 2.5.1 Data exclusion

Following data collection, we excluded 21 participants who either experienced technical issues or showed insufficient response, defined as a standard deviation of less than 5 tokens across their individual WTP ratings. The final sample, therefore, included 165 participants.

#### 2.5.2 Extraction of visual features

Visual features were extracted using the open-source Python tool (Schwenzow et al., 2021), which automatically extracted 109 video-based variables from raw video files, derived from both frame-level and video-level information. This tool is available at https://github.com/JasperLS/Understanding_Videos_at_Scale/blob/master/Understanding_Videos_at_Scale.ipynb.

To prevent overfitting and ensure model robustness, we implemented a systematic feature-selection protocol prior to executing any predictive analyses. First, we restricted our scope to 48 video-level features, retaining variables that provided a single measure per video, either directly or through aggregation across the entire video. Second, we removed facial emotion features, as the detection algorithm produced missing values across a substantial portion of the commercial set. Third, we excluded specific color-space channels, maintaining only overall colorfulness. Finally, to avoid multicollinearity, we dropped one of two features that had a correlation higher than 0.7 with each other. This pipeline reduced the original set to 10 low-level visual features used in subsequent analyses (see Table 1 for descriptions). The table detailing which specific features were eliminated and retained, as well as the feature correlation matrix, can be found in Supplementary Appendices A3, A4.

**Table 1:** Visual features used for the analysis.

Feature number	Feature	Description
1–2	Colorfulness	Colorfulness, as defined by Hasler and Süsstrunk (2003), is expressed as a percentage and measured based on the opponent color space. It is calculated using the standard deviation and mean of the red-green and yellow-blue differences. In our analysis, we used both the mean and variance of colorfulness values.
3–4	Saturation	Saturation taken from the 𝑆 channel in HSV color space in %. We used both the mean and variance saturation values.
5–6	Luminosity	Luminosity taken from the 𝑉 channel in HSV color space in %. We used both the mean and variance luminosity values.
7	Faces ratio	Number of frames with faces divided by the number of frames in %
8	Object human area	Average share of pixels belonging to people in %
9	Quality	The average quality across all frames, measured using the variance of the Laplacian (Pech-Pacheco et al., 2000).
10	Scene frequency	Total number of scenes divided by video length in seconds.

All descriptions are taken from Schwenzow et al. (2021).

#### 2.5.3 Extraction of auditory features

For each commercial, we extracted 12 auditory features, both from the time and frequency domains, such as zero-crossing rate and spectral bandwidth. To do so, we used Librosa library in Python (McFee et al., 2015). We also calculated the RMSE using a different GitHub repository (Velardo, 2020).^1^ After removing highly correlated features (*r* > 0.7), 9 auditory features remained. Table 2 describes the nine auditory features we used for analysis after the feature selection procedure. The table detailing which specific features were eliminated and retained, as well as the feature correlation matrix, can be found in Supplementary Appendices A5, A6.

**Table 2:** Auditory features used for the analysis.

Feature number	Feature	Description
1–2	Zero crossing rate (ZCR)	The rate of sign-changes of the signal during the duration of a particular frame. We used both the mean and variance values
3–4	Root mean-square energy (RMSE)	The root mean square of the signal’s amplitude values within a frame. We used both the mean and variance RMSE values
5–6	Tempo	The overall estimated tempo of a track in beats per minute (BPM). We used both the mean and variance values
7	Band energy ratio	Quantifies the energy distribution across different frequency bands within the audio signal.
8–9	Spectral bandwidth	The average width of the signal’s frequency spectrum. We used both the mean and variance values.

#### 2.5.4 Prediction labels

We averaged the WTP and *Liking* values for each commercial across all participants. In other words, for each commercial we obtained a single mean score for each subjective measure (WTP and *Liking*) across participants. The rationale for this approach is that each commercial has the same set of visual and auditory features across participants, and, therefore, we aim to capture the overall effect on the average pool of participants as a proxy to the effect at the population level. This aggregation approach aligns with our objective of identifying how low-level features drive population-level preferences, rather than modeling individual heterogeneity. By focusing on the average effect, we can more effectively predict the average effect of low-level features on preferences.

#### 2.5.5 Controlling for commercial identity

In studies like ours, where participants rated multiple items, it is important to account for individual differences in ratings across different video commercials. Some participants may consistently give higher or lower scores across all commercials, which could lead to a potential bias. To test that the variability in ratings was truly driven by the commercials themselves, and not by individual rating tendencies, we performed a linear regression with subjective rankings (either *Liking* or WTP) as the dependent variable and commercial ID as the independent variable. This analysis was conducted on the complete dataset, meaning that instead of using the average *Liking* or WTP per commercial, we included all individual ratings. To account for repeated measures (i.e., multiple ratings from the same participant), we clustered the standard errors by participant.

#### 2.5.6 Prediction procedures

For all prediction procedures, we conducted the same analysis, once for the WTP values and once for the *Liking* ratings. For each of the subjective rankings in each model, we conducted three prediction attempts: (1) Using only low-level visual features. (2) Using only low-level auditory features. (3) Using the combination of both visual and auditory features. A description of the analysis stages is presented in Figure 2.

**Figure 2:**
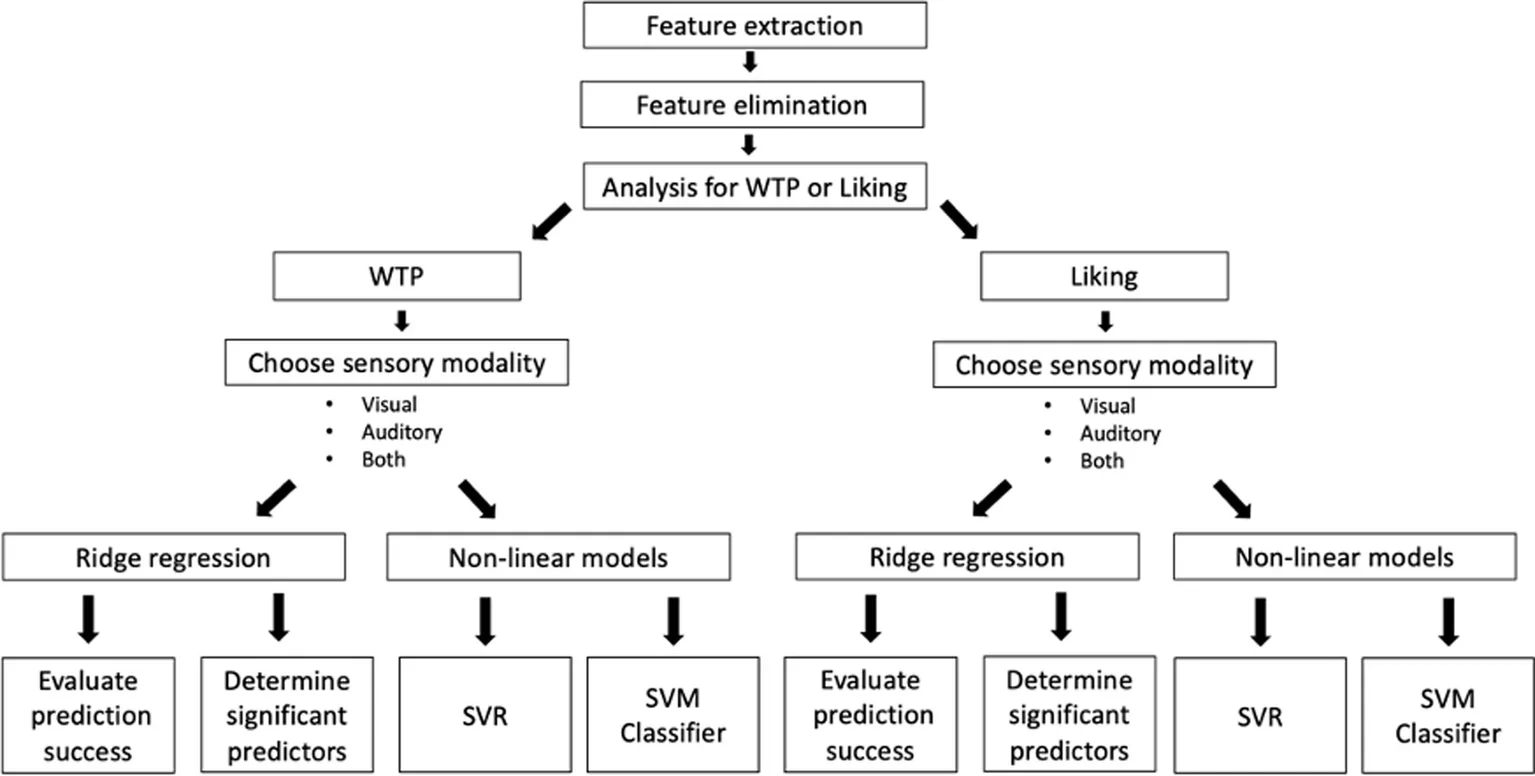
Analysis stages. First, we extracted low-level features from the video commercials. We then conducted a feature elimination process (for more details, see the Methods section). We conducted a separate analysis for WTP and *Liking*. We first conducted the analysis using only visual features, then using only auditory features, and finally, we performed the analysis using a combination of both. We conducted a prediction model using Ridge regression and tested it using an out-of-sample prediction (LOO). We computed beta averages across all LOO iterations. In a parallel analysis, we also examined the data using nonlinear models [Support Vector Regression (SVR) and Model (SVM)].

#### 2.5.7 Out-of-sample predictions

First, we examined how well a linear model with the selected low-level features can predict out-of-sample either the WTP or *Liking* values. To achieve this, we employed ridge regression with a Leave-One-Out (LOO) Cross-Validation in Python. We chose this model as it is able to incorporate regularization, reducing the risk of overfitting. Throughout this section, index j denotes the specific LOO iteration (i.e., which of the 100 commercials was left out as the test set), while index i denotes an individual commercial within the training set of that iteration. In each iteration j (j = 1, …, 100), the dataset was split into a training set of 99 commercials and a test set consisting of the single left-out commercial. Both features and outcome measures were standardized using only the training set parameters to prevent data leakage:

$$X_{i}^{\prime }=\frac{X_{i}-\mu_{\text{train}}}{\sigma_{\text{train}}},y_{i}^{\prime }=\frac{y_{i}-\mu_{\text{train}}}{\sigma_{\text{train}}}$$

Then, we fit the ridge regression on the standardized training data:

$${\hat{\beta }}^{\left(j\right)}=\text{argmin}\_\beta \;[\;\Sigma \_\{i\in \text{train}\}\;{\left(y_{i}^{\prime }-X_{i}^{\prime }\beta -b\right)}^{2}+\alpha {\left\|\beta \right\|}^{2}]$$

where ${\hat{\beta }}^{\left(j\right)}$ is the vector of regression coefficients, ${\left(y_{i}^{\prime }-X_{i}^{\prime }\beta -b\right)}^{2}$ is the squared error between the observed standardized outcome $y_{i}^{\prime }$ and the value predicted by the model $X_{i}^{\prime },$ b is the intercept, and α controls the strength of the L2 (ridge) penalty. The regularization parameter α was selected dynamically within each training fold via an internal 5-fold cross-validation grid search (α∈ {0.1,1,10}). Finally, the standardized rating for the left-out test commercial was predicted as:

$${\hat{y}}_{j}^{\prime }=X_{j}^{\prime }{\hat{\beta }}^{\left(j\right)}+{\hat{b}}^{\left(j\right)}$$

where ${\hat{y}}_{j}^{\prime }$ is the predicted value (Liking or WTP), $X_{j}^{\prime }$ represents the standardized low-level features of the test commercial using $\mu_{\text{train}}$ and $\sigma_{\text{train}}$ , ${\hat{\beta }}^{\left(j\right)}$ is the fitted coefficient vector, and ${\hat{b}}^{\left(j\right)}$ s the fitted intercept. Model performance was evaluated across all iterations using $R^{2}$ , Mean Absolute Error (MAE), and Mean Squared Error (MSE).

#### 2.5.8 Feature importance

To extract the most important features contributing to each model, we computed the average regression coefficient for each feature across all 100 Leave-One-Out (LOO) iterations:

$${\bar{\beta }}_{\mathrm{obs}}=\frac{1}{100}\;\sum_{j=1}^{100}{\hat{\beta }}^{\left(j\right)}$$

This gives a single “observed” coefficient per feature $({\bar{\beta }}_{\mathrm{obs}}),$ summarizing its average contribution across all 100 iterations.

To obtain *p*-values for the features in the model, we conducted a permutation test with *Q* = 500 iterations to construct an empirical null distribution. In each permutation q, the outcome values (either Liking or WTP) were randomly shuffled and at the end we calculated a permutation-specific average coefficient for each feature. We then compared the real, observed coefficient $\left({\bar{\beta }}_{\mathrm{obs}}\right)$ to this null distribution to assess whether the observed coefficient significantly differed from chance. The two-tailed empirical *p*-value for each feature was calculated as the proportion of permuted null coefficients whose absolute magnitude was equal to or greater than the absolute value of the observed coefficient:

$$p_{\text{feature}}=\frac{1}{5000}|\{q:|{\bar{\beta }}^{\left(q\right)}|\geq |{\bar{\beta }}_{\mathrm{obs}}|\}|$$

where ${\bar{\beta }}^{\left(q\right)}$ is the average coefficient obtained in permutation q (one of the 5,000 times) and $|\{q:|{\bar{\beta }}^{\left(q\right)}|\geq |{\bar{\beta }}_{\mathrm{obs}}|\}|$ is the count of how many of those 5,000 permutations had a coefficient equal to or greater than the actual coefficient. We applied a False Discovery Rate (FDR) correction (Benjamini and Hochberg, 1995) for all the *p*-values of the coefficients and then determined which regressors were significant.

#### 2.5.9 Testing with non-linear models

To explore whether we could predict the subjective rankings using a non-linear model, we employed a support vector regression (SVR) for either the WTP values or the *Liking* ratings. The SVR estimates the function $\left(f(X)=w^{T}\varphi (X)+b\right)$ by minimizing:

$$\min\_(w,b)\;\frac{1}{2}{\left\|w\right\|}^{2}+C\cdot \sum_{i=1}^{N}\max(0,|y_{i}-f(X_{i})|-\varepsilon )$$

where f(X) is the prediction (Liking or WTP), w is the weight vector, φ(X) is the mapping function transforming inputs into a higher-dimensional feature space, b is the intercept, ${\left\|w\right\|}^{2}$ is the squared norm of the weight vector, ε defines a margin of tolerance around the observed value, $\max(0,|y_{i}-f(X_{i})|-\varepsilon )$ is the penalty for predictions that fall *outside* this margin and C is the regularization parameter.

To make sure that our results are not biased because of a specific split of the data into training and test sets (80% train and 20% test), we conducted 1,000 different splits of training and test sets and report the average metrics (MSE, MAE, and R^2^). For each train and test split, we performed hyperparameter tuning via randomized search and tested several kernels [linear, radial basis function (RBF), polynomial, and sigmoid]. The search space included the regularization parameter C, the margin ε, the kernel coefficient γ, and, for the polynomial kernel, the degree parameter.

#### 2.5.10 Calculating prediction accuracies

As regression models do not directly provide prediction accuracies, we also employed a Support Vector Model (SVM) classifier for either the WTP values or the *Liking* ratings. We divided the WTPs and the *Liking* ratings into high and low values using a median split. The SVM model was trained on the low-level features to classify whether a commercial would be ranked high or low. To make sure that our results were not biased because of a specific split of the data into training and test sets (80% train and 20% test), we conducted 1,000 different splits of training and test sets and averaged the results. For each train and test split, we performed the hyperparameter tuning described above. We also extracted the mean accuracy, sensitivity, specificity, precision, and negative predictive value of these 1,000 splits to provide a robust estimate of model performance. Similarly, using the same binarization method and splitting procedure, we repeated the classification procedure but using a logistic regression model defined as:

$$\ln(\frac{p_{i}\;}{1-p_{i}\;})=z_{i}=w^{T}X_{i}+b$$

Where $\ln(\frac{p_{i}\;}{1-p_{i}\;})\;$ is the log-odds, the natural log of the probability that commercial i is high-value divided by the probability that it is not. And $z_{i}$ is the weighted sum of the commercial’s standardized features.
Which gives the conditional probability:
$$P(y\_\mathrm{bi}n_{i}=1\;|\;X_{i})=1/(1+\exp(-(w^{T}X_{i}+b)))$$

Where $P(y\_\mathrm{bi}n_{i}=1\;|\;X_{i})$ represents the conditional probability that commercial i belongs to the high-value rating (1), $X_{i}$ is the vector of low-level features of commercial i , $w^{T}$ is the weight vector, $\left(w^{T}X_{i}+b\right)$ is the linear combination of inputs and weights, b is the intercept, and exp. is the exponential function.

#### 2.5.11 Validation against random chance

Finally, to ensure that the results from the SVM and logistic regressions were not due to chance and to confirm the validity of our model’s predictive power, we repeated the same steps using shuffled data of the subjective rankings. That is, we randomly shuffled the subjective rankings (either WTPs or *Liking*) and repeated the prediction procedure.

#### 2.5.12 Predicting YouTube metrics

We gathered publicly available metrics from YouTube, regarding each of the commercials. Note that at the time of data collection, not all 100 YouTube links were still available. Therefore, we used only 91 available commercials for this analysis. These YouTube metrics served as a proxy for commercial effectiveness at the population-level. The YouTube metrics we collected were the number of likes, views, and comments. We adjusted each metric to account for the duration the commercial had been online, giving less weight to older commercials by multiplying each metric of each commercial with the ratio between the number of days the oldest commercial has been online divided by the number of days that that commercial was online. We collected all metrics on the same day, and all commercials were online for more than 6 months. Then, we divided each specific metric in each commercial by the sum of that same metric in all other commercials. Thereafter, we performed a PCA on the relative metrics and used the summed score of the two first components, which together explained above 75% of the variance, as the aggregate score for each commercial. These aggregate scores were finally converted into 91 ordered rankings of the commercials’ success in YouTube, based on the order of scores. We used these overall rankings as labels for prediction.

## 3 Results

### 3.1 Descriptive statistics and rating variability

First, we examined the distribution of *Liking* and WTP values across participants. Figure 3A (*Liking*) and Figure 3B (WTP) describe the average values across participants for each commercial (M_Lik_ = 3.48, Sd_Lik_ = 0.58; M_WTP_ = 39.9, Med_WTP_ = 39.45, Sd_WTP_ = 5.86). To assess variability across commercials, we conducted Levene’s tests for equality of variances. The results indicated significantly unequal variances across commercials (*p* < 0.01).

**Figure 3:**
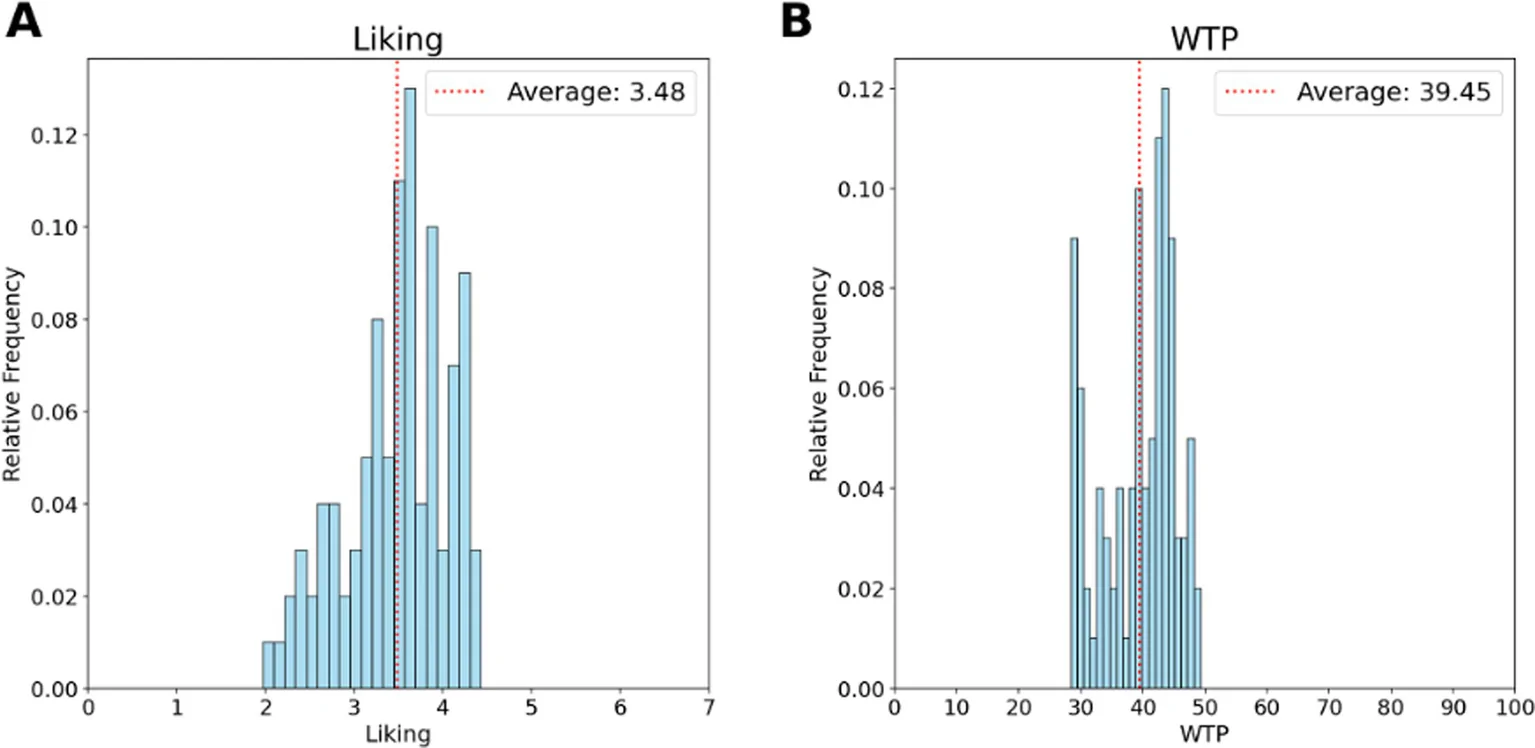
The relative frequency of subjective rankings of commercials across participants. **(A)** *Liking*. **(B)** WTP. The red dotted line represents the average of the distribution.

In addition, we assessed whether participants’ ratings were distinct enough across commercials and exhibited sufficient variability. Specifically, we tested if there was a linear relationship between subjective rankings (*Liking*/WTP) and commercial ID (a unique number given to each video commercial). As shown in Figure 4, both *Liking* and WTP ratings were broadly distributed across commercials. Linear regressions confirmed that commercial ID explained little to no variance in either outcome (*Liking*: *R*^2^ = 0.002, *p* > 0.05; WTP: *R*^2^ = 0.00, *p* > 0.05). This indicates that subjective evaluations varied across commercials and were suitable for feature-based prediction.

**Figure 4:**
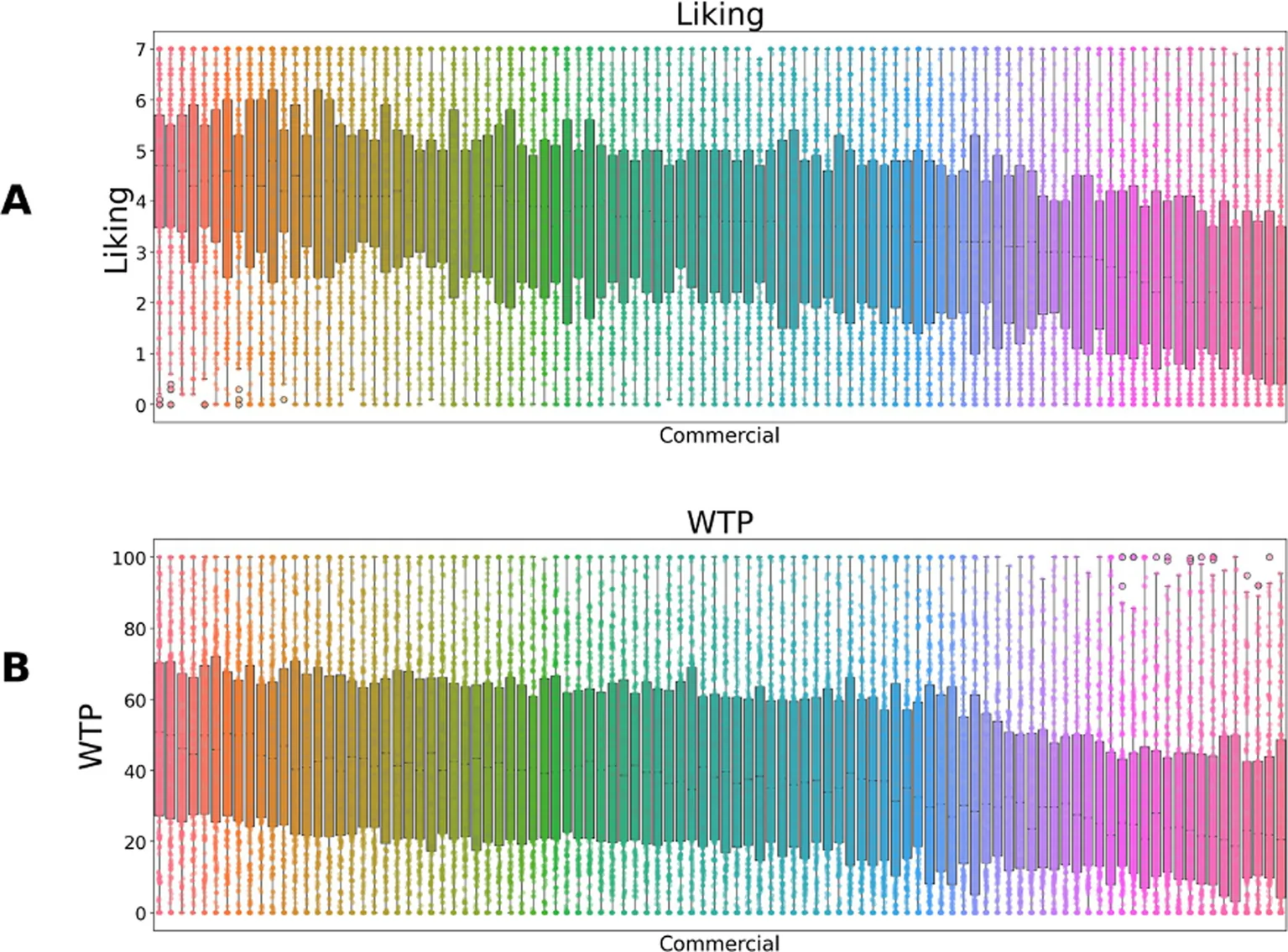
Values of subjective rankings. Value ratings for *Liking* **(A)** and WTP **(B)** for all commercials are represented as boxplots. Each boxplot represents the distribution of the subjective ranking for a specific commercial. It includes median, quartiles, and the whiskers (inter-quartile range). In addition, the figure includes a strip plot which represents every individual value on the boxplot.

### 3.2 Prediction of liking and WTP from Low-level visual features

We first tested whether low-level visual features could predict average *Liking* and WTP ratings across commercials using a ridge regression with a Leave-One-Out Cross-Validation.

Figures 5A,B describes the correlation between the average (across all the LOO iterations) predicted values and the actual measured values. Out-of-sample prediction significantly exceeded permutation-based null models. *Liking* ratings had an R^2^ of 0.33 (MSE = 0.700; MAE = 0.664), and lower (but not negligible) WTP values had an R^2^ of 0.17 (MSE = 0.859; MAE = 0.740). This suggests that low-level visual features can predict (out-of-sample) the average *Liking* ratings and WTP values that participants assign to commercials after watching them.

**Figure 5:**
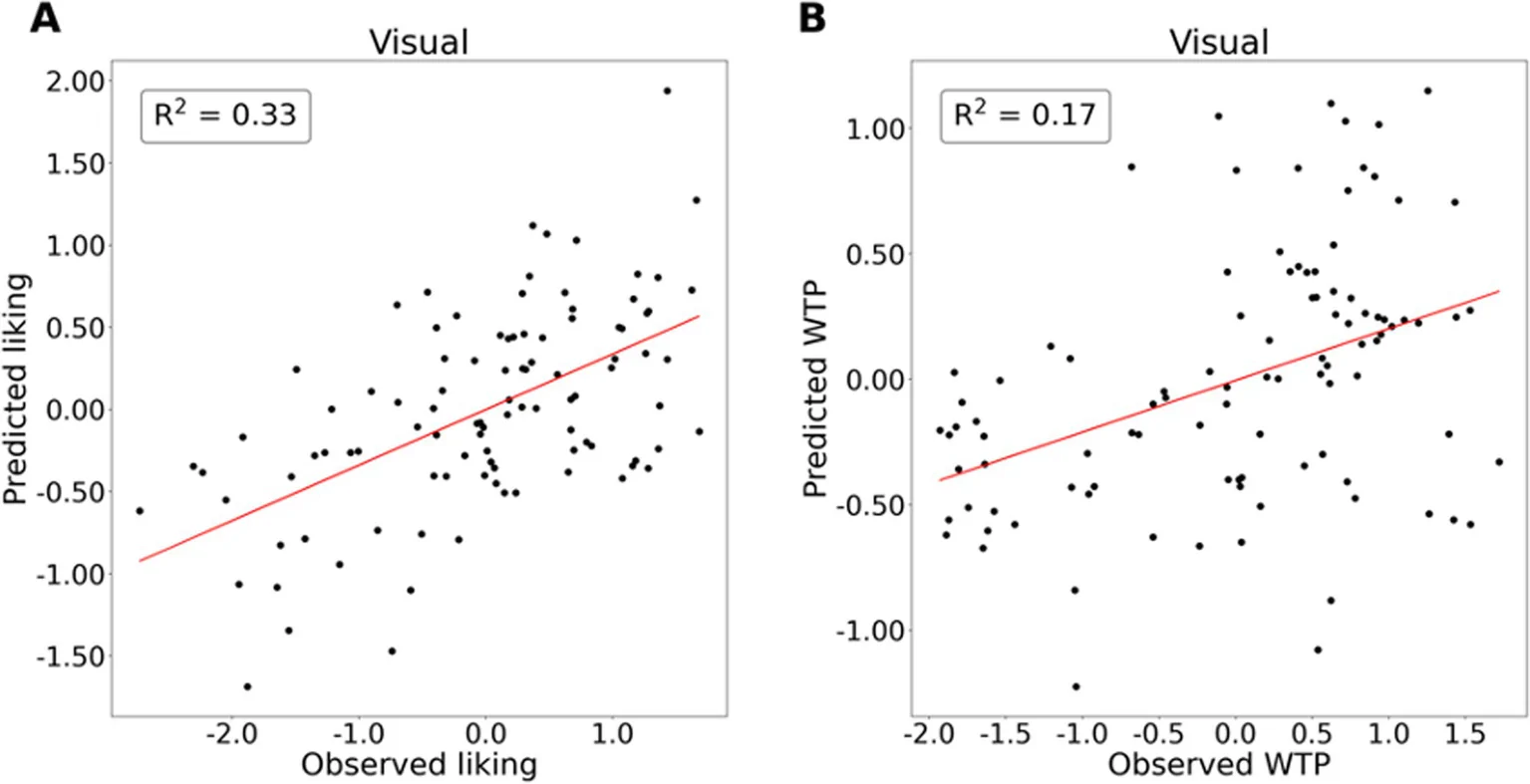
Prediction results for low-level visual features. The comparison of observed vs. predicted values for *Liking* **(A)** and WTP **(B)**. Each dot represents the average value for a commercial across participants. The red line represents the best-fitted line. *R*^2^ represents the average goodness-of-fit across all LOO iterations. Note, values are normalized.

### 3.3 Which visual features show the strongest predictive associations?

Thereafter, we averaged the beta coefficients from all the LOO regression iterations and calculated the *p*-values for all coefficients by comparing them to a null distribution obtained from 5,000 permutations using shuffled labels. Figure 6 describes the values of the coefficients of all features for predicting *Liking* and WTP. Our analysis reveals that scene frequency showed the largest standardized coefficients in predicting both *Liking* and WTP (β_Lik_ = 0.368, p_Lik_ < 0.001; β_WTP_ = 0.453, p_WTP_ < 0.001). This suggests a strong positive correlation between the number of scenes per second and commercial rankings, particularly since the clip lengths were tightly controlled at roughly 6 s (standard deviation of 0.42 s). Additional features, including mean luminosity and mean saturation, also exhibited statistically reliable coefficients for *Liking*. Specifically, mean luminosity showed a positive relationship (*β* = 0.312, *p* < 0.01) and mean saturation showed a moderate but significant negative effect (*β* = −0.319, *p* < 0.01). These results indicate that multiple visual features contribute differentially to prediction performance across outcome measures.

**Figure 6:**
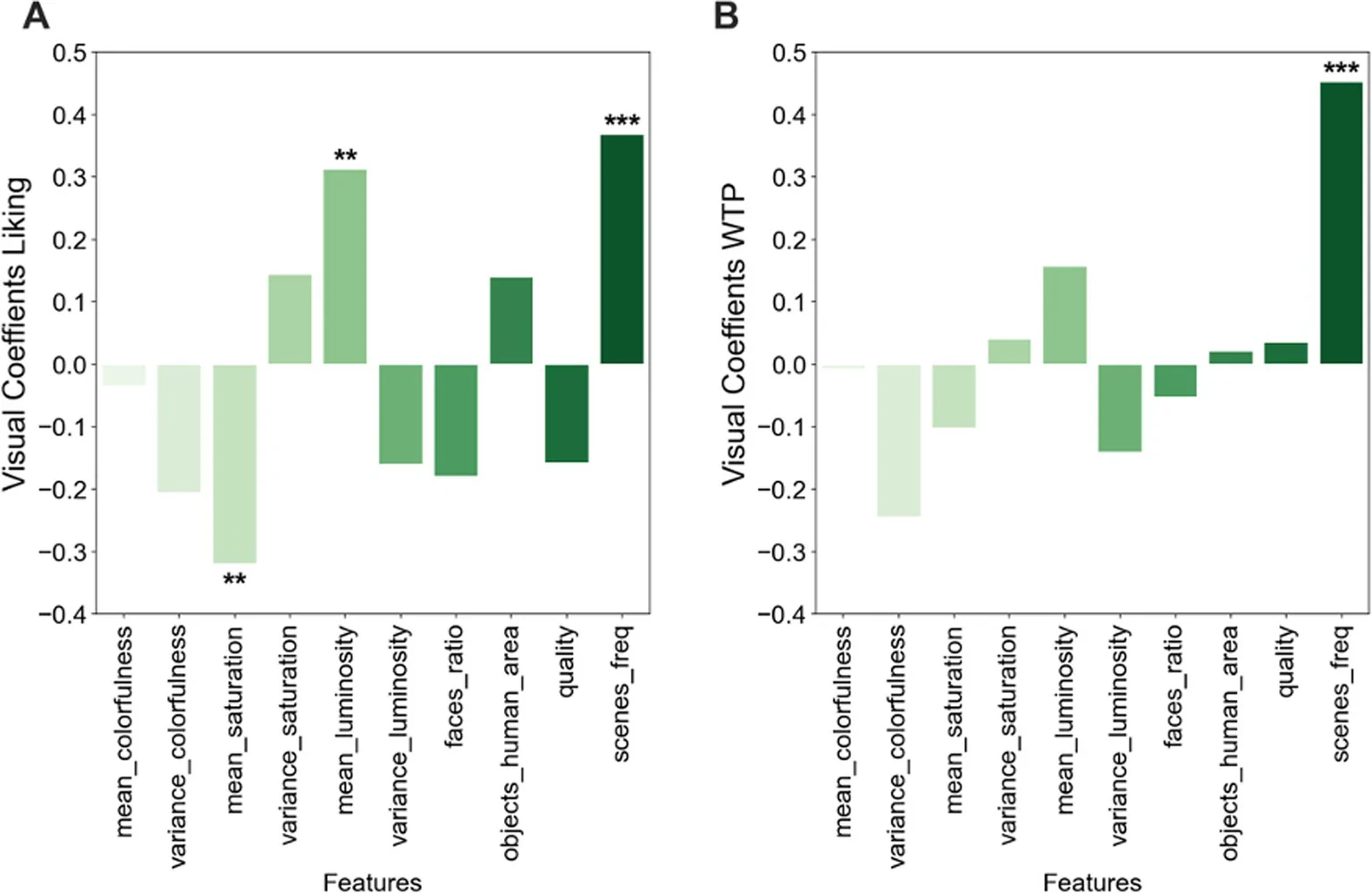
Low-level visual influential features. The most influential low-level visual features (x-axis) on subjective rankings of commercials—*Liking* **(A)** and WTP **(B)**. The y-axis represents the values of the average coefficients from the LOO regressions. ***p < 0.001, **p < 0.01, *p < 0.05, all FDR corrected.

As a robustness check, we repeated the same analysis without any feature selection, using the 48 aggregates and video-level extracted features. None of the coefficients reached significance before and after FDR correction (see Supplementary Appendix A14 for details)

### 3.4 Low-level auditory features can also predict subjective rankings of commercials

As for the low-level visual features, we examined whether auditory features alone could predict subjective evaluations (WTP and *Liking* values). We extracted 9 auditory features, both from the time and frequency domains, such as zero-crossing rate, root mean square energy (RMSE), and spectral bandwidth. We conducted the exact same prediction procedure as we did for the low-level visual features but using the extracted low-level auditory features instead (see methods for details).

Auditory-only models showed a high correlation between the predicted and actual values for the WTP values (*R*^2^ = 0.31, MSE = 0.711, MAE = 0.685) (Figure 7A) and less so (but not negligible) for the *Liking* ratings (*R*^2^ = 0.10, MSE = 0.932, MAE = 0.787) (Figure 7B). These results suggest that low-level auditory features can also predict the average *Liking* ratings and the WTP values that participants assign to commercials after watching them.

**Figure 7:**
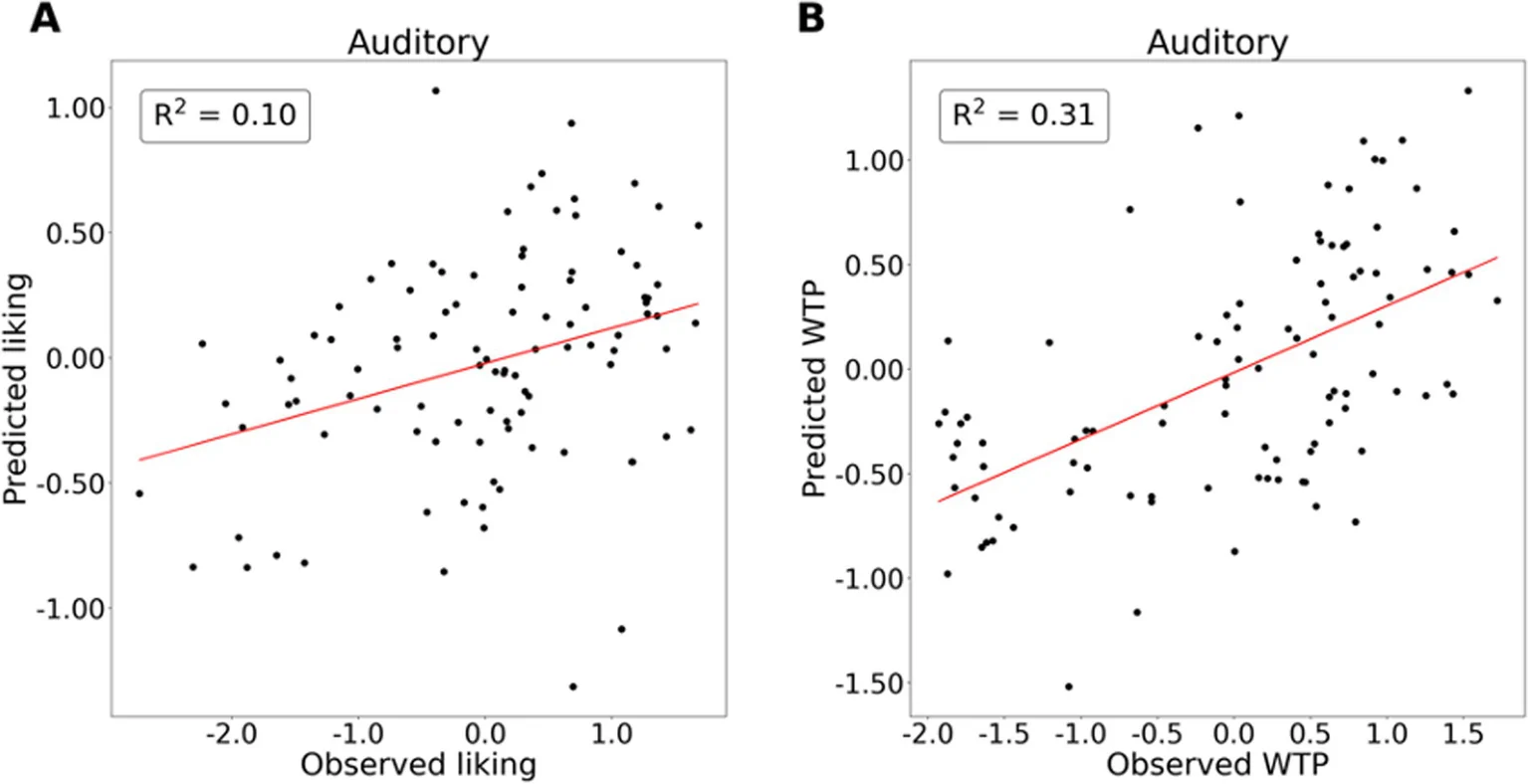
Prediction results for low-level auditory features. The correlation between the predicted values from the model and the actual values measured for l*iking* **(A)** and WTP **(B)**. Each dot represents the average value for a commercial. The red line represents the best-fitted line. *R*^2^ represents the average goodness-of-fit across all LOO iterations. Note, values are normalized.

### 3.5 Which auditory features show the strongest predictive associations?

As with the visual features, we averaged the beta coefficients from all the LOO regression iterations, and calculated *p*-values for the coefficients by comparing them to a null distribution obtained from 5,000 permutations using shuffled labels. Figure 8 describes the coefficients of the auditory features. Note, that the most influential features in both regressions are the average zero crossing rate (ZCR) with a large negative coefficient (β_Lik_ = −0.398, p_Lik_ < 0.01; β_WTP_ = −0.372, p_WTP_ < 0.01), and its variance with a large positive coefficient (β_Lik_ = 0.405, p_Lik_ < 0.01; β_WTP_ = 0.439, p_WTP_ < 0.001). Zero-crossing rate (mean and variance) exhibited the largest absolute coefficients across both outcome variables. These features consistently contributed to prediction performance in the auditory-only models.

**Figure 8:**
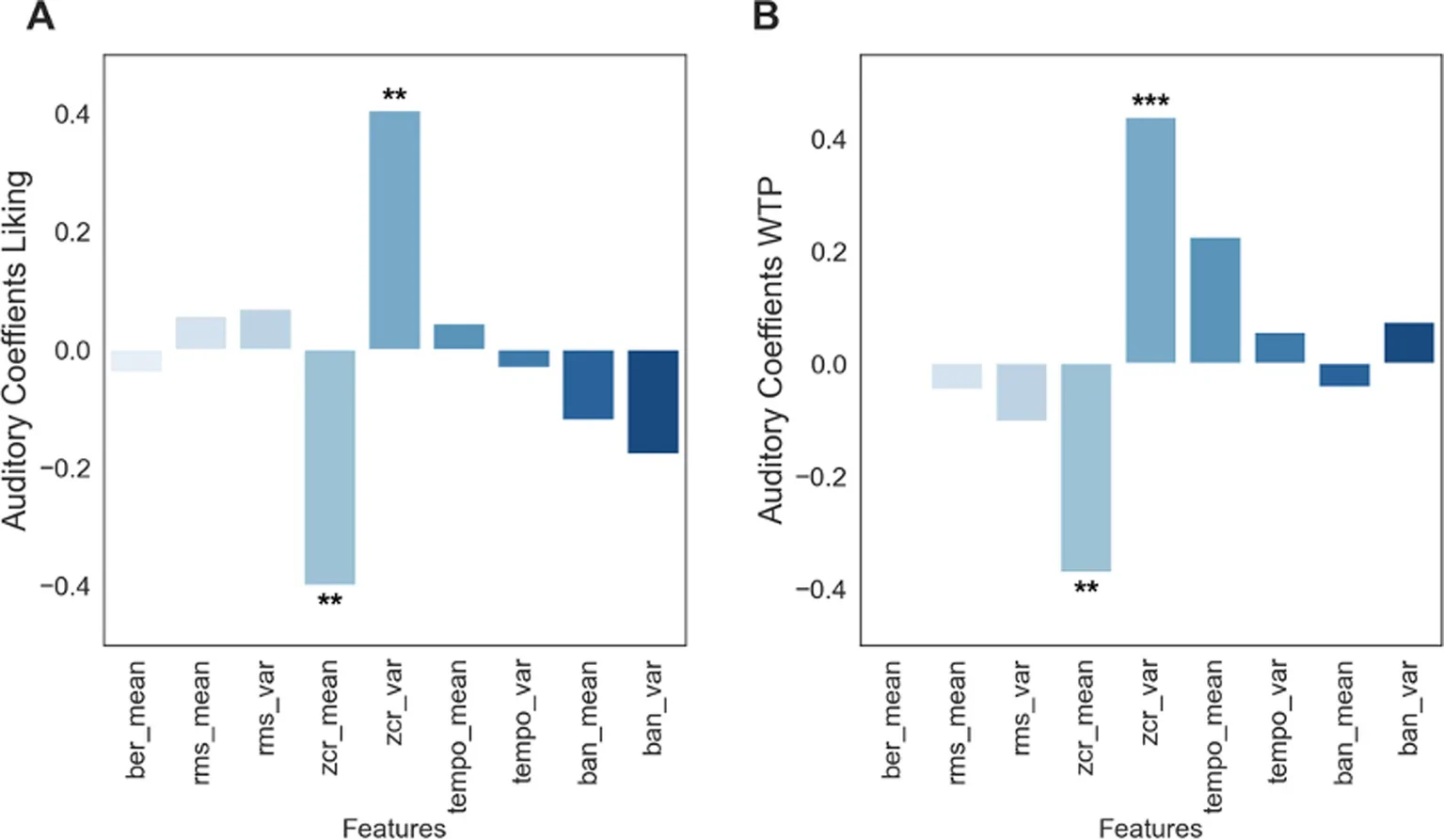
Low-level auditory influential features. The most influential low-level auditory features (x-axis) on subjective rankings of commercials—*Liking* **(A)** and WTP **(B)**. The y-axis represents the values of the average coefficients from the LOO regression iterations. ***p < 0.001, **p < 0.01, *p < 0.05, all FDR corrected.

### 3.6 Multimodal prediction: combining visual and auditory features

Having established that visual and auditory features independently predict subjective evaluations, we tested whether combining both modalities improve prediction. We repeated the prediction procedure, but now using both the visual and auditory features. As shown in Figure 9, multimodal models improved prediction accuracy for WTP relative to single-modality models with an *R*^2^ = 0.37 (MSE = 0.650 and MAE = 0.614). While the prediction of *Liking* remained comparable to the visual-only model, with an *R*^2^ = 0.33 (MSE = 0.696 and MAE = 0.673). This indicates that auditory features provide information that is complementary, particularly for WTP.

**Figure 9:**
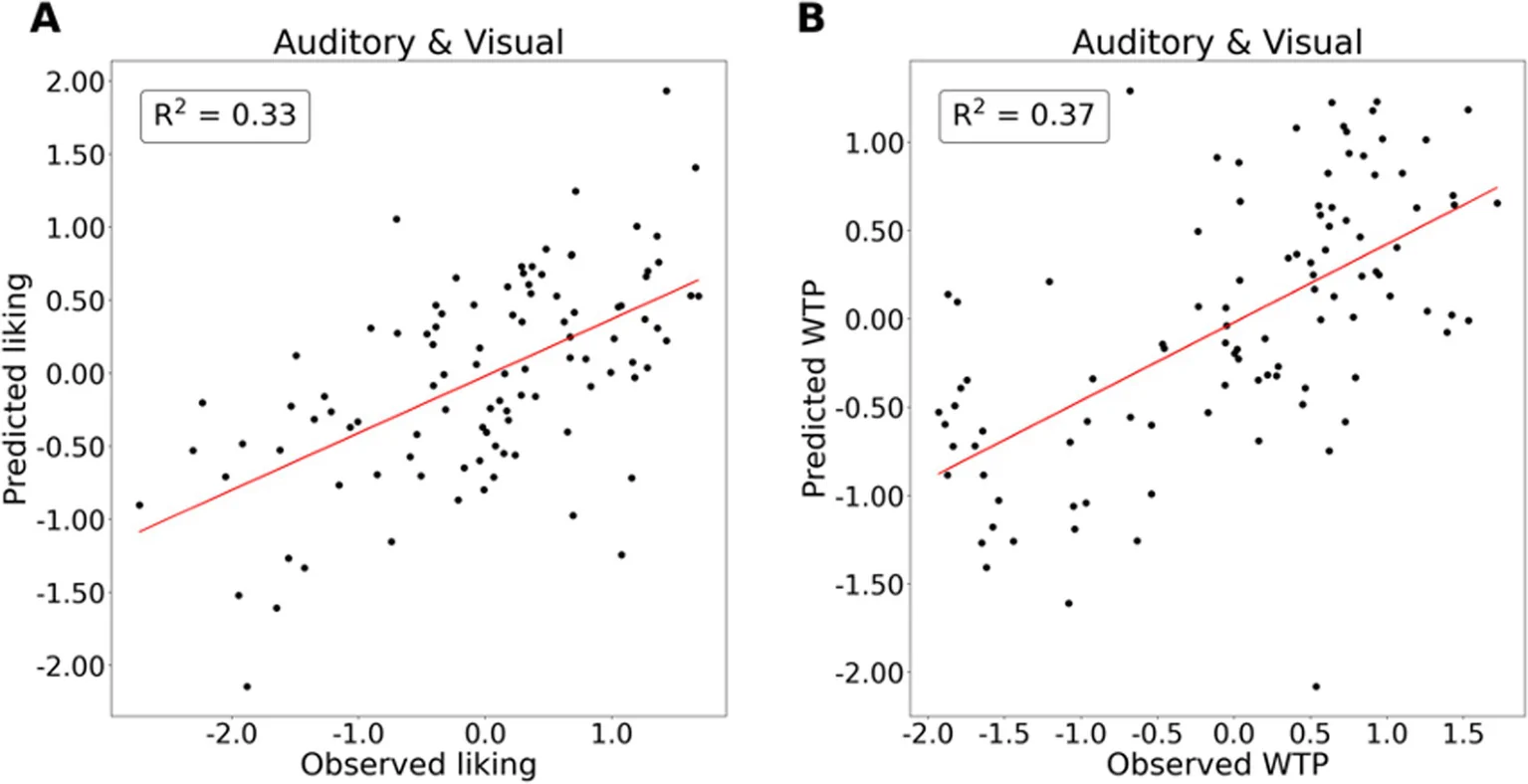
Prediction results for the combined low-level visual and auditory features. The correlation between the predicted values from the model and the actual values measured for *Liking* **(A)** and WTP **(B)**. Each dot represents the average value for a commercial. The red line represents the best fitted line. *R*^2^ represents the average goodness-of-fit across all LOO iterations.

### 3.7 Which features show the strongest predictive associations in the multimodal models?

Following the same procedure as in the single-modality models, the combined models (Figure 10) showed that scene frequency remained the strongest predictor for both outcomes (β_Lik_ = 0.312, p_Lik_ < 0.05; β_WTP_ = 0.358, p_WTP_ < 0.01), while auditory ZCR variability contributed uniquely to WTP (β_WTP_ = 0.386, p_WTP_ < 0.01). Both features maintained the significance observed in our single-modality models. These results suggest that while visual dynamics dominate affective responses (*Liking*), valuation judgments (WTP) integrate both visual and auditory cues.

**Figure 10:**
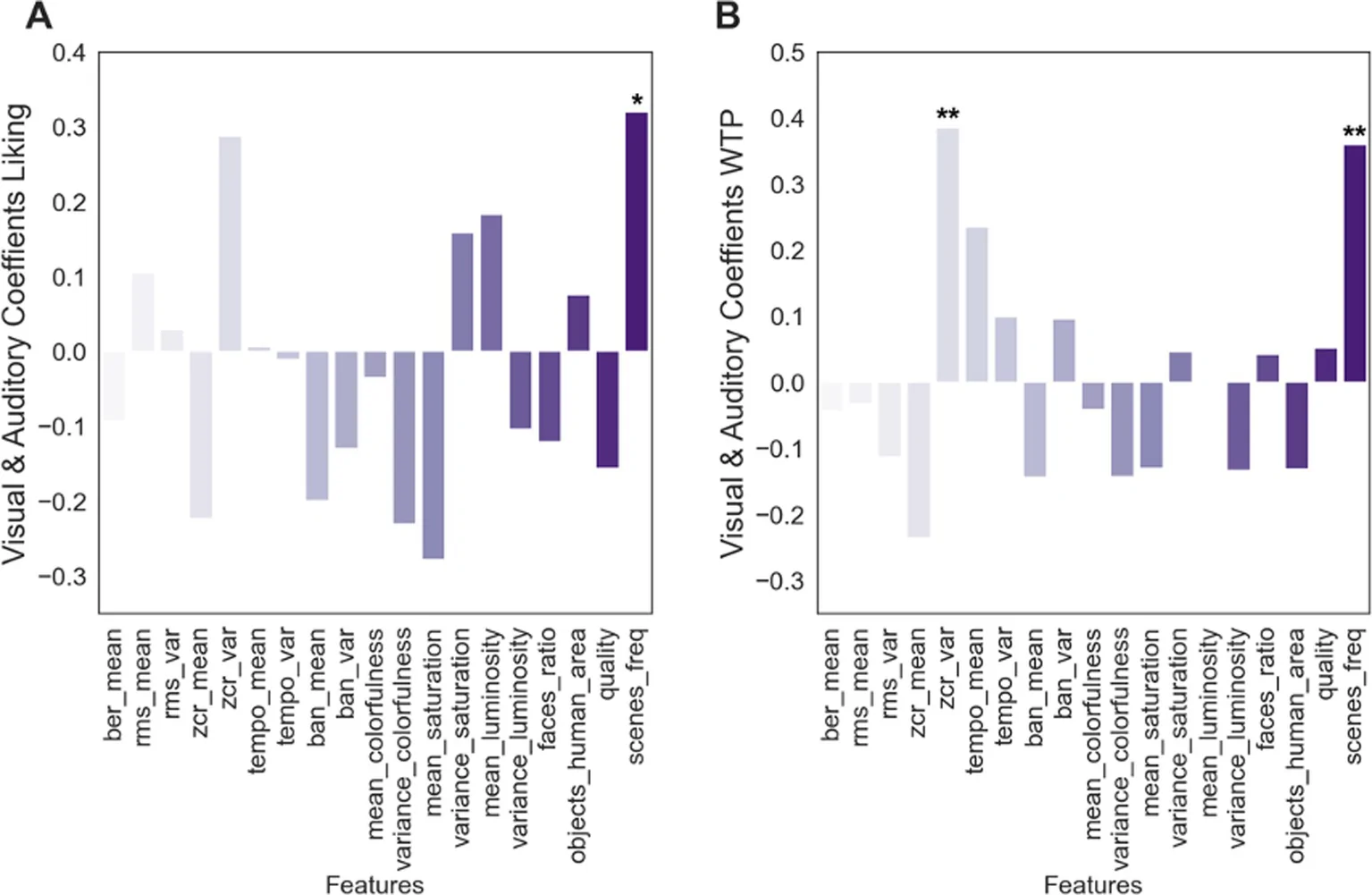
Low-level visual and auditory influential features. The most influential features (x-axis) on subjective rankings of commercials—l*iking* **(A)** and WTP **(B)** when using both visual and auditory low-level features. The y-axis represents the values of the average coefficients from the LOO regressions. ***p < 0.001, **p < 0.01, *p < 0.05, all FDR corrected.

### 3.8 Using a non-linear model for prediction

Up until now, we used only a linear model in our prediction attempts. Next, we wanted to examine if a non-linear model would provide better prediction results by using an SVR model. Consistent with our linear approach, we trained SVR models using visual-only, auditory-only, and combined feature sets. Across all outcomes, the non-linear Radial Basis Function (RBF) emerged as the dominant kernel (see Supplementary Appendix for details). Model comparisons revealed that RBF-based SVR outperformed linear models in predicting out-of-sample WTP.

Figure 11 describes the correlations between the observed and predicted values for the models using only low-level visual features. For *Liking* ratings, nonlinear models offered little improvement over the linear model, with comparable performance metrics (*R*^2^ = 0.33, MSE = 0.634, MAE = 0.606). For WTP values, however, the nonlinear model significantly outperformed the linear version (*R*^2^ = 0.52, MSE = 0.448, MAE = 0.538).

**Figure 11:**
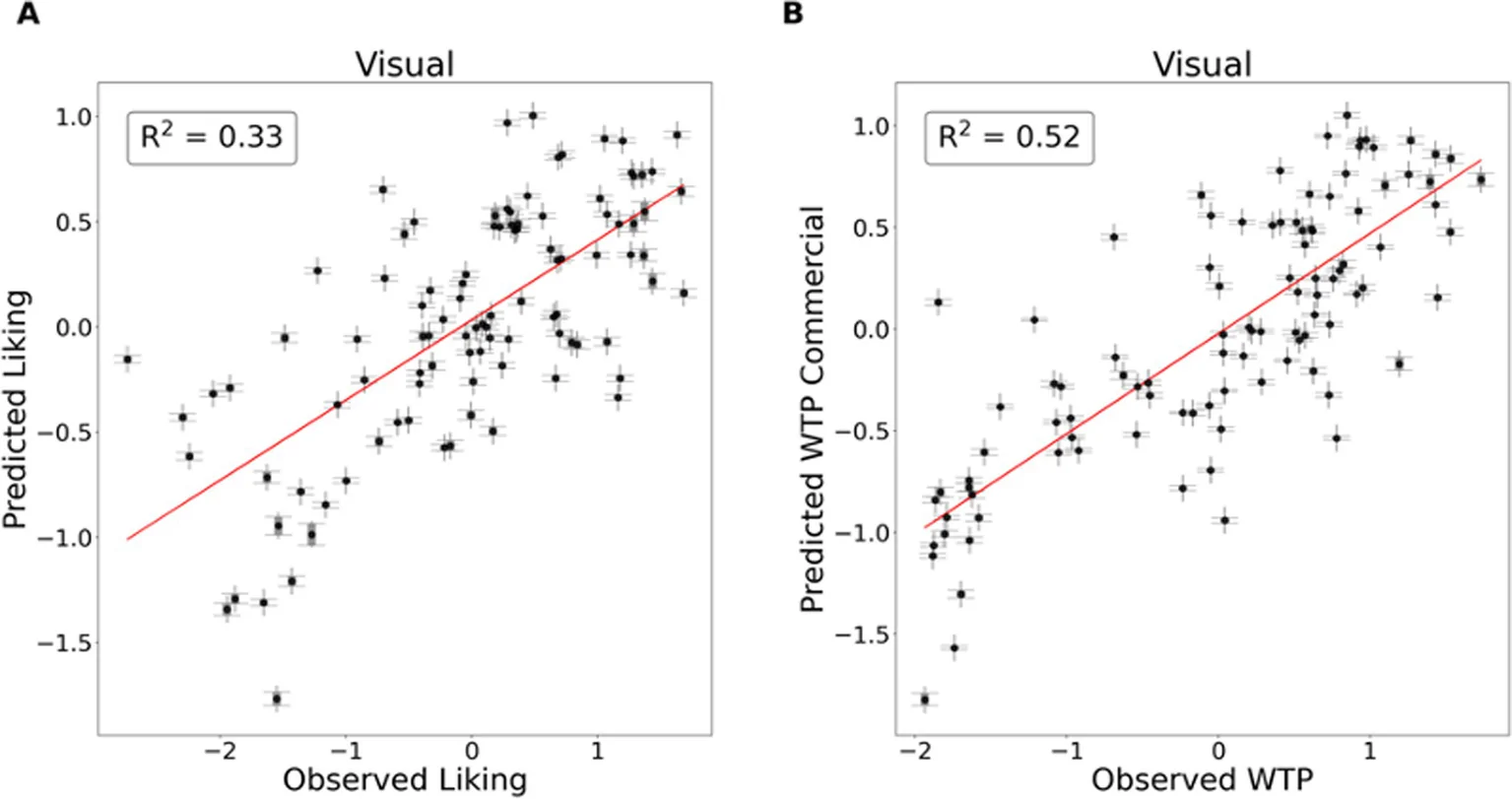
Prediction results of the SVR model using low-level visual features only. **(A,B)** The correlation between the model’s predicted and observed values for *Liking* and WTP, respectively. Each dot represents a single commercial, with values corresponding to the mean predicted and mean observed scores across multiple randomized cross-validation iterations. The red line indicates the best-fitting linear regression. Error bars represent the standard error of the mean (SEM). The R^2^ value reflects the model’s goodness-of-fit.

Figure 12 illustrates the correlation between the predicted and observed values for *Liking* and WTP using the SVR model considering only the auditory low-level features. For *Liking* ratings, this model achieved an *R*^2^ = 0.07 (MSE = 0.886 and MAE = 0.762). For WTP values, the model achieved an *R*^2^ = 0.39 (MSE = 0.590 and MAE = 0.559). This indicates that using the nonlinear model improved only WTP predictions, while *Liking* predictions seem to be better captured by the linear model.

**Figure 12:**
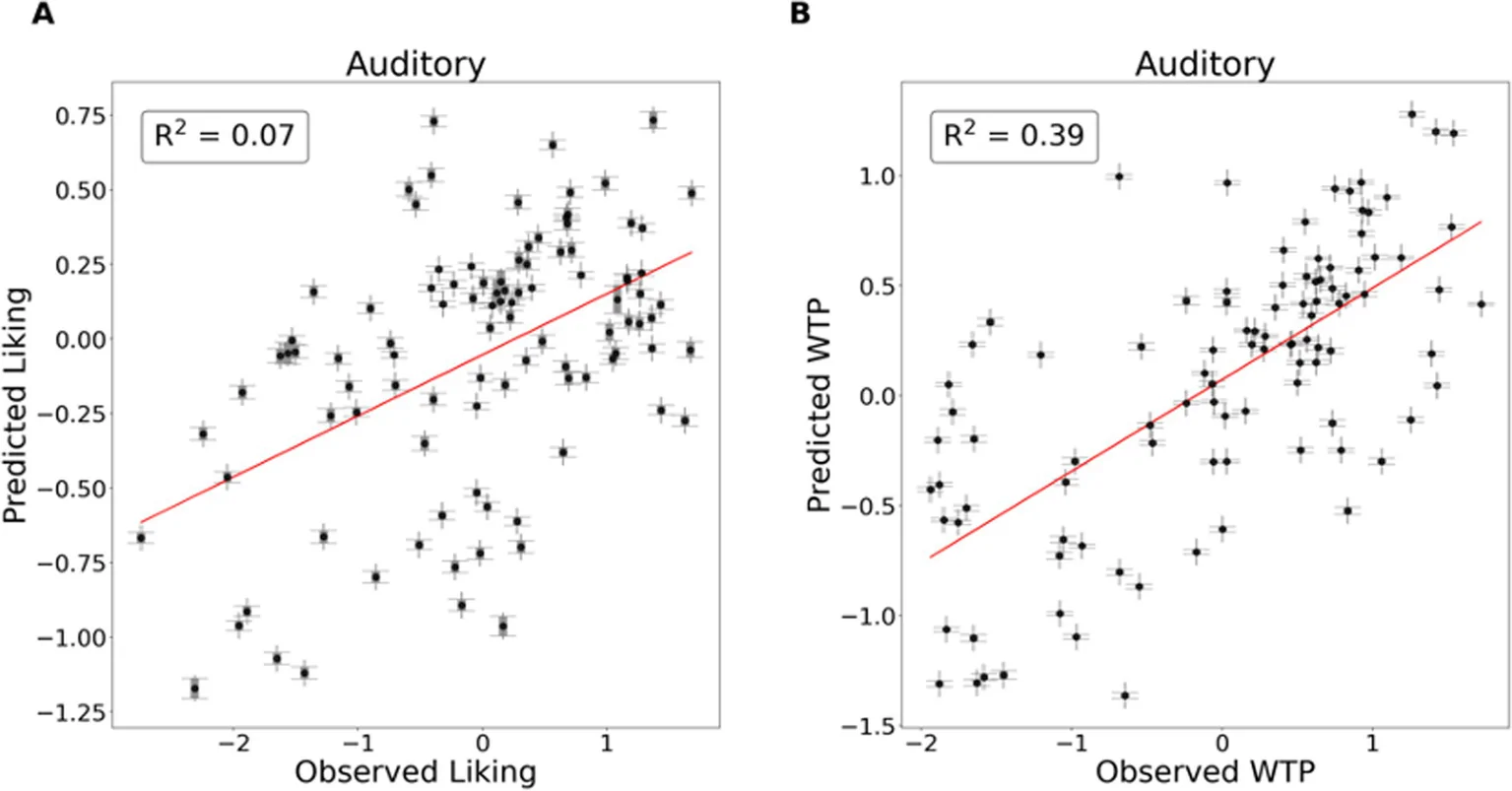
Prediction results of the SVR model using low-level auditory features only. **(A,B)** The correlation between the model’s predicted and observed values for Liking and WTP, respectively. Each dot represents a single commercial, with values corresponding to the mean predicted and mean observed scores across multiple randomized cross-validation iterations. The red line indicates the best-fitting linear regression. Error bars represent the standard error of the mean (SEM). The R^2^ value reflects the model’s goodness-of-fit.

Figure 13 illustrates the relationship between the predicted and observed values for either *Liking* (Figure 13A) and WTP (Figure 13B) using the SVR models with both visual and auditory low-level features. For *Liking* ratings, this model achieved an *R*^2^ = 0.39 (MSE = 0.587 and MAE = 0.604). Importantly, for WTP values, the model achieved a very high *R*^2^ = 0.66 (MSE = 0.334 and MAE = 0.424). Importantly, note that both nonlinear models outperformed the linear models when including both visual and auditory low-level features.

**Figure 13:**
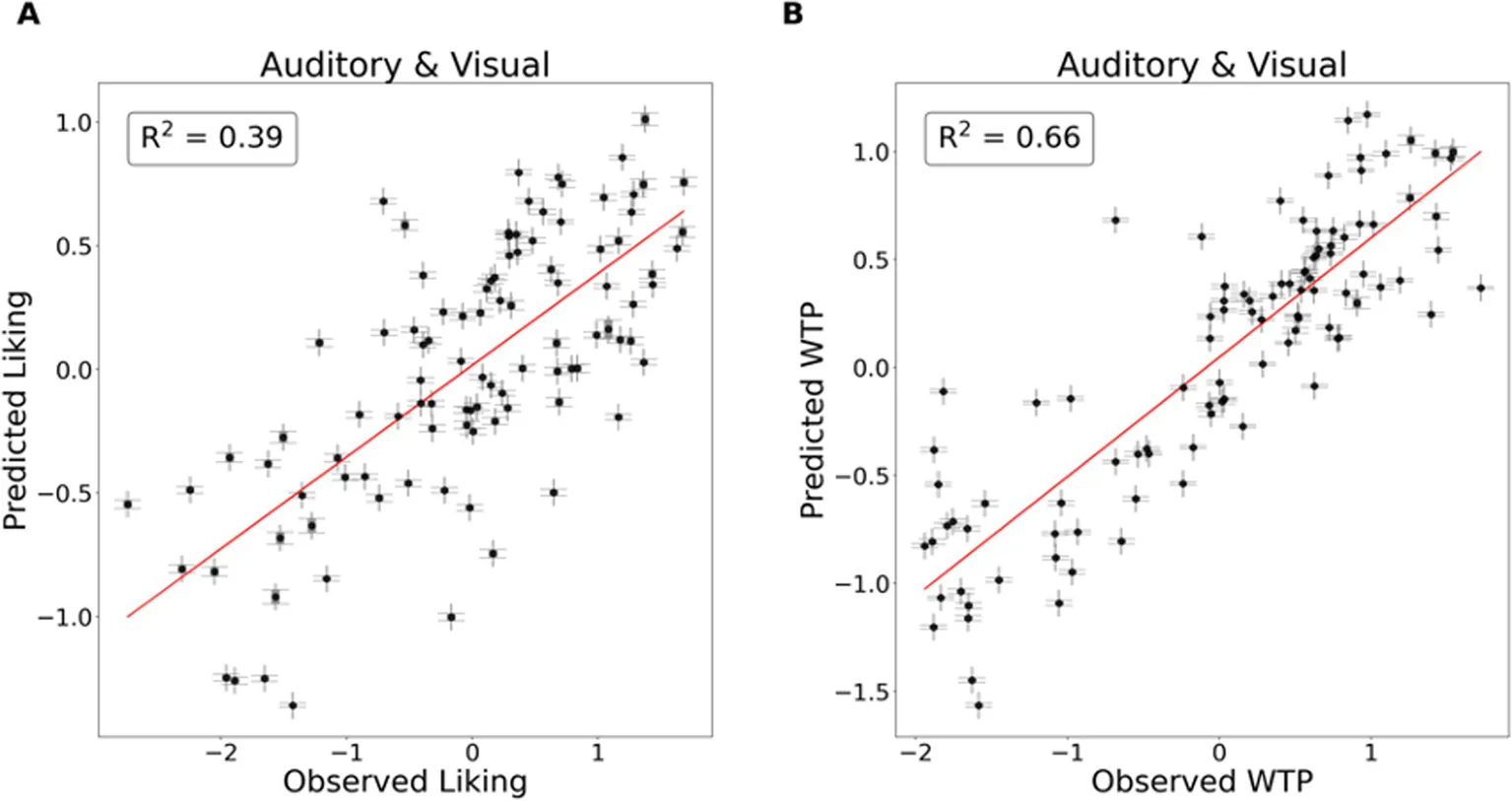
Prediction results of the SVR model using both low-level visual and auditory features. Panels **(A,B)** show the correlation between the model’s predicted and observed values for Liking and WTP, respectively. Each dot represents a single commercial, with values corresponding to the mean predicted and mean observed scores across multiple randomized cross-validation iterations (SEM is used for the error bars). The red line indicates the best-fitting linear regression. Error bars represent the standard error of the mean (SEM). The *R*^2^ value reflects the model’s overall goodness-of-fit.

Overall, across all analyses, multimodal models integrating visual and auditory features consistently outperformed single-modality versions, particularly for WTP. Prediction performance was generally higher for WTP than for *Liking*, and nonlinear models improved WTP prediction across all feature sets. A concise overview of all linear and non-linear model performances is provided in Table 3. Overall, nonlinear, and multimodal models demonstrated significant performance increments, especially for WTP.

**Table 3:** Summary of the goodness-of-fit (R2) of the prediction models.

Outcome	Features	Linear R^2^	Nonlinear (SVR) R^2^	Key finding
*Liking*	Visual	0.33	0.33	Relationship is fully captured linearly
*Liking*	Auditory	**0.10**	0.07	Weak predictive power
*Liking*	Visual + auditory	0.33	**0.39**	Modest improvement for multimodal nonlinear model
WTP	Visual	0.17	**0.52**	Strong improvement nonlinear visual model
WTP	Auditory	0.31	**0.39**	Moderate nonlinear improvement
WTP	Visual + auditory	0.37	**0.66**	Largest improvement multimodal nonlinear model

Values in bold indicate the higher *R*² value (best-performing model between linear and nonlinear) for each outcome and feature combination row.

### 3.9 Classification task

To complement continuous prediction analyses, we conducted binary classification of high versus low *Liking* and WTP. Both SVM and logistic regression classifiers achieved accuracies well above chance (61–80%), with higher accuracy for WTP than for *Liking*. Multimodal models again performed best, reinforcing the conclusion that combining visual and auditory cues improves prediction robustness (for a detailed description of the classification results, see Supplementary Appendix).

### 3.10 Predicting YouTube metrics

Next, we wanted to examine if we can use the low-level features to predict the success of the commercials at the population level. To do so, we aimed to predict an aggregate YouTube engagement metric as an external validity check and a proxy for the success of the commercials at the population level (see methods for details). Using the same procedure as for the *Liking* and WTP models, we predicted each commercial’s aggregate YouTube score from low-level visual and auditory features. We tested an out-of-sample prediction using Ridge regression with only low-level visual features (Figure 14A), achieving an *R*^2^ = 0.03 (MSE = 1.00, MAE = 0.833). Importantly, the corresponding non-linear SVR model (Figure 14B) significantly outperformed the linear model, achieving a much higher *R*^2^ = 0.25 (MSE = 0.707, MAE = 0.684). In contrast, models with auditory only features (Figures 14C,D) were better captured by the linear model with an *R*^2^ = 0.30 (MSE = 0.718, MAE = 0.683), while the non-linear SVR model performed worse with an *R*^2^ = 0.07 (MSE = 0.898 and MAE = 0.742).

**Figure 14:**
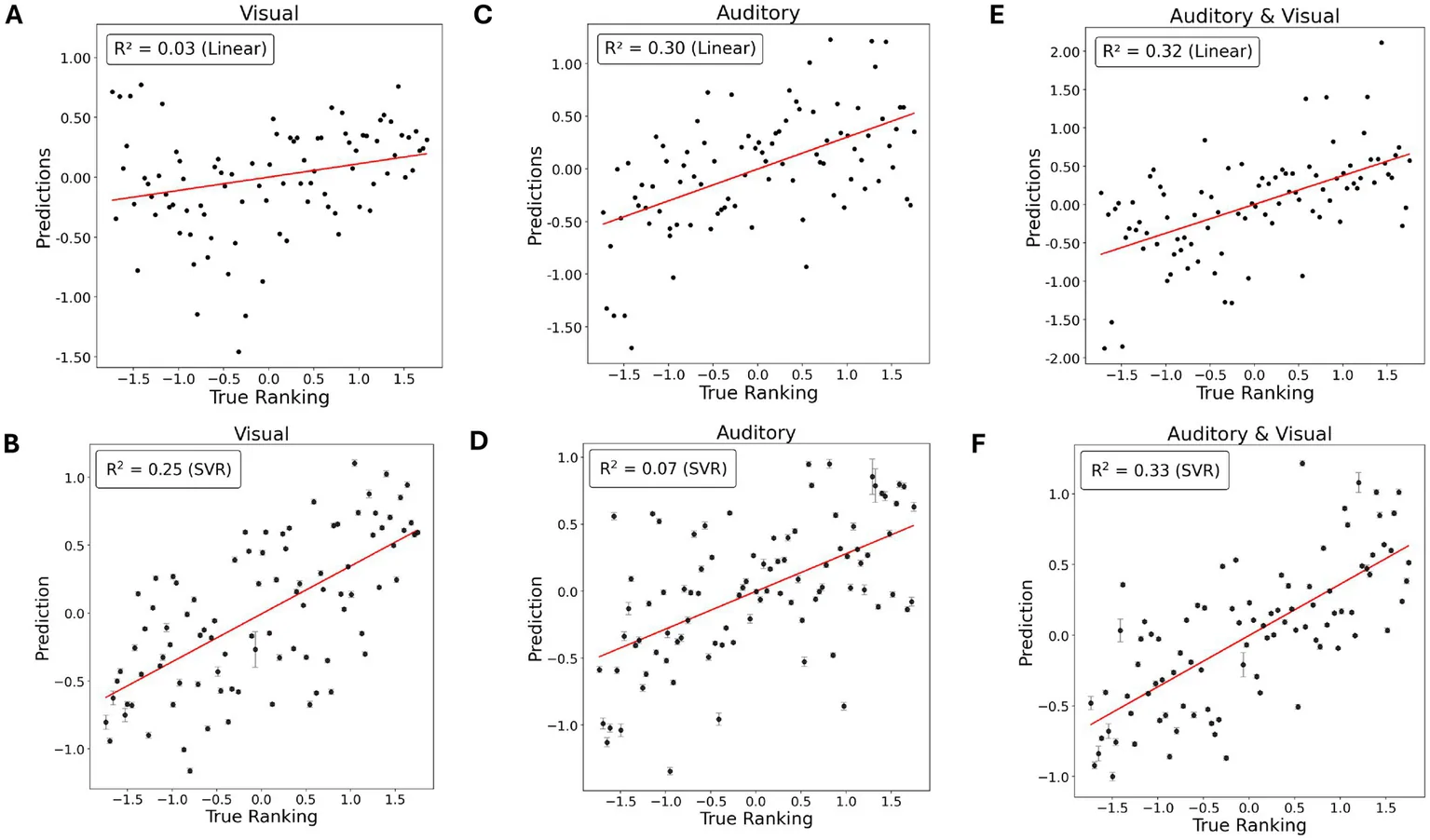
Prediction results of YouTube success using low-level features. **(A,B)** The correlation between the predicted and observed YouTube scores when using only visual features with Ridge regression **(A)** and SVR **(B)**. **(C,D)** The corresponding results when using only auditory features with Ridge regression **(C)** and SVR **(D)**. Panels **(E,F)** show the results when combining both visual and auditory features with Ridge regression **(E)** and SVR **(F)**. Each dot represents a single commercial, with values corresponding to the mean predicted and observed scores across cross-validation iterations. The red line indicates the best-fitting linear regression. *R*^2^ represents the overall goodness-of-fit of each model. Error bars represent the standard error of the mean (SEM).

Again, the most robust results emerged from the combined models. Integrating both sensory features resulted in the highest predictive power (Figures 14E,F). The Ridge regression model reached a slightly better result than the auditory only model with an *R*^2^ = 0.32 (MSE = 0.698, MAE = 0.674), while the SVR had significantly higher performance compared to the visual only model with an *R*^2^ = 0.33 (MSE = 0.644, MAE = 0.642). These results indicate that low-level auditory and visual features significantly and reliably predict valuations at the population level, which reinforces the generalizability of our sensory-based predictive framework

## 4 Discussion

In this study, we examined whether low-level visual and auditory features can predict consumers’ subjective evaluations of video commercials measured as ad likability and WTP for the advertised product. Our first main finding is that models combining both modalities consistently outperformed unimodal models, particularly for WTP, where multimodal models achieved the highest explained variance (*R*^2^ = 0.37 for linear and *R*^2^ = 0.66 for non-linear models). This indicates that auditory and visual information provide complementary value in shaping consumer preferences. Additionally, the higher predictive power of non-linear models, especially for WTP, suggests that the relationship between early sensory information and subjective value depends on complex interactions among low-level perceptual features that are better captured by non-linear models.

Visual elements alone significantly contributed to the prediction of both *Liking* and WTP with *R*^2^ values between 0.17 and 0.52. This predictive power supports the idea that low-level visual features play an important role in influencing preferences and perceived value of video commercials. Our findings align with previous research showing that low-level visual features can predict esthetic preferences of different item categories and art (Oren et al., 2020; Iigaya et al., 2021).

In addition to the visual features, we showed that auditory features alone also contributed significantly to the prediction of subjective preferences. Our results are consistent with studies showing that auditory low-level features can predict consumers’ preferences, such as song popularity (Zangerle et al., 2019; Martinez et al., 2021). Here, we expanded this notion to video commercials.

Moreover, both the non-linear and linear models successfully predicted the engagement ranking metric as measured from YouTube (*R*^2^ ≥ 0.32). This finding suggests that beyond predicting subjective preferences of participants in the lab, low-level visual and auditory features can also serve as reliable indicators of video effectiveness at the population level.

In line with this, it was recently shown that sales increased when the visual presence of the product overlapped with areas of high engagement in pixel-level heatmaps (Yang J. et al., 2025).

### 4.1 Theoretical implications

Our results extend advertising and consumer behavior research in several ways. First, they demonstrate that low-level perceptual properties of dynamic audiovisual commercials systematically influence subjective evaluation, moving beyond work focused on static images and behavioral metrics, such as likes, shares, and click-through rates (Trunfio and Rossi, 2021).

Second, the findings support the Elaboration Likelihood Model (Petty and Cacioppo, 1986) by highlighting the role of sensory information in consumer valuation. In short-form advertising, low-level auditory and visual features may provide a perceptual basis that influences the final valuation, particularly in environments characterized by limited attention and high information density. Third, although visual-only models generally showed stronger predictive power than auditory-only models, consistent with visual dominance in adults (Spence, 2009; Robinson et al., 2016), the superior performance of the combined modalities models demonstrates a complementary effect of auditory and visual information, supporting the role of multisensory integration in shaping perception and preference (Opoku-Baah et al., 2021; Williams et al., 2022). Fourth, non-linear models outperformed the linear models in predicting WTP across all feature sets (visual-only, auditory-only, and combined) and for *Liking* scores in the combined models. This suggests that the interactions between visual and auditory low-level features with subjective evaluations is non-linear and complex. Fifth, by also predicting a YouTube aggregate engagement metric the results suggest that low-level features can predict at a population level. This aligns with previous research demonstrating that visual and auditory features are strong predictors of real-world outcomes, such a YouTube song popularity (Lee and Tu, 2025).

### 4.2 Interpretation of key predictors

Among the visual features examined, we found that scene frequency emerged as the most robust visual predictor for both *Liking* and WTP. Scene frequency was derived using the open-source software PySceneDetect (AlMousa et al., 2018; Shou et al., 2018), which was implemented in the tool developed by Schwenzow et al. (2021). This software captures changes in hue, saturation, and luminosity between frames, which is closely linked to motion contrast. We found a positive association between scene frequency and subjective rankings. This suggests that dynamic visual changes enhance attentional capture, consistent with prior research linking attention allocation to choice and valuation (Krajbich et al., 2012; Milosavljevic et al., 2012; Tavares et al., 2017).

Despite the finding in prior studies that abrupt transitions can reduce comprehension or enjoyment (Kim et al., 2014; Liu et al., 2018), our results suggest that commercials with higher scene frequency increases subjective evaluations. One explanation for these discrepancies is that the relationship between scene frequency and preference is not strictly linear but may follow a U-shaped pattern, where moderate levels of scene frequency enhance engagement, while very low or very high levels could be detrimental. In our study, the observed range of scene frequencies likely fell within this beneficial zone, explaining the positive association. We also found that lower saturation and higher luminosity increase *Liking* ratings, supporting the role of visual saliency in shaping affective responses and perceived value (Labrecque and Milne, 2012; Milosavljevic et al., 2012; Fu et al., 2024).

Among the auditory features examined, ZCR (zero-crossing rate) and its variance, were the strongest auditory predictors. ZCR is typically linked to the presence of voice in segments, with lower values indicating voiced frames and higher values indicating unvoiced segments (Jalil et al., 2013). Lower ZCR values (more voiced frames) and higher ZCR variability consistently predicted higher *Liking* and WTP. This aligns with research suggesting that viewers prefer voiced content (Barnes and Wang, 2024) and that clear voice quality enhances positive affect (Barnes, 2024). Despite potential overlap between ZCR and scene frequency, the low correlation between them (*r* = 0.15) indicates that visual and auditory features provide complementary information. Additionally, variation in ZCR may reflect changes in voice energy and pitch dynamics, which have been shown to capture viewer attention and drive subjective evaluations (Peterson et al., 1995; Guo et al., 2014).

Importantly, even in the models combining both modalities, scene frequency and ZCR variance were consistently the most important predictors, highlighting the complementary nature of visual and auditory cues. The stability of these features across both unimodal and combined models suggests a reliable and persistent association between these specific low-level features and viewers’ subjective evaluations.

### 4.3 Practical implications for marketers and advertising agencies

Our findings offer several implications for practitioners in short-form video advertising. First, they suggest that effectiveness is influenced not only by message content or branding elements, but also by sensory design choices. Adjusting scene pacing, luminosity, and color saturation, alongside optimizing vocal clarity and dynamic variation, may systematically enhance ad likability and perceived product value.

Second, the improved performance of multimodal models implies that the interaction between auditory and visual features should be treated as a strategic design variable. Rather than optimizing visual and auditory elements independently, agencies may benefit from aligning changes across modalities to reinforce attention and affect.

Third, the successful application of machine learning models highlights their potential as diagnostic tools that complement traditional evaluation approaches, helping identify sensory configurations that are more likely to increase both ad likability and WTP.

### 4.4 Limitations

This study has several limitations. First, our sample consisted exclusively of university undergraduates from a single institution, which may restrict the generalizability of our behavioral and valuation findings to broader, demographically diverse consumer populations. Second, we did not measure participants’ familiarity with the commercials, prior product use, or existing brand relationships, which may have influenced their evaluations. These factors are known to influence purchase intentions and WTP and may have biased evaluations if participants relied on past experiences rather than the commercials themselves (Kim and Chung, 2012). However, we mitigated these concerns by focusing our analysis on average scores across participants rather than individual-level data. Furthermore, by using well-known, established brands, we ensured a relatively stable baseline of brand familiarity and equity. Third, participants viewed a relatively large number of commercials within a single session. While this extended exposure can lead to fatigue, such a state may have increased the relative weight of low-level sensory features in the evaluation process. Importantly, we counterbalanced the stimulus order across participants, which mitigates the risk that fatigue systematically biased the evaluation of specific products or commercials. Fourth, the stimulus set was largely restricted to supermarket products, which limits the generalizability of our findings to other product categories. Fifth, low-level features were aggregated across entire videos, overlooking frame-by-frame dynamics, which could play an important role in shaping attention and valuation. Finally, our stimulus set was limited to commercials retrieved from YouTube. YouTube was selected because tools such as yt-dlp, an established open-source command-line tool for media retrieval, allow systematic access to video content and relevant public metadata, whereas platforms such as TikTok and Instagram have more restrictive API access. However, this reliance on YouTube may limit the generalizability of our findings to other advertising environments.

### 4.5 Directions for future research

Future research should explicitly measure brand familiarity, prior usage, and brand attitudes to disentangle sensory-driven effects from pre-existing preferences. In addition, incorporating temporal analyses of audiovisual dynamics could provide deeper insight into how specific moments within commercials drive attention and valuation. Extending this framework to a broader range of product categories would further enhance external validity.

## 5 Conclusion

In this study we demonstrate that low-level visual and auditory features predict consumer preferences for video commercials in the lab and at the population level. We found that predictive models combining both auditory and visual features significantly outperform unimodal approaches, suggesting that consumers’ preferences are driven by the combined influence of multiple low-level sensory information rather than by isolated inputs. Particularly, when non-linear approaches are considered, our findings highlight the importance of this multisensory integration in early-stage preference formation. Our results relate low-level perceptual processing and consumer valuation which offers both theoretical insight and practical guidance for the design of short-form video advertising.

## Data Availability

The datasets presented in this study can be found in online repositories. The names of the repository/repositories and accession number(s) can be found GitHub at: https://github.com/v-parada/commercial-preferences-low-level-features-data.git.

## Footnotes

1
https://github.com/musikalkemist/AudioSignalProcessingForML

## References

[ref1] AlMousa M. Benlamri R. Khoury R. (2018). NLP-enriched automatic video segmentation 2018 6th International Conference on Multimedia Computing and Systems (ICMCS) 1–6. doi: 10.1109/ICMCS.2018.8525880

[ref2] Areni C. S. Kim D. (1994). The influence of in-store lighting on consumers’ examination of merchandise in a wine store. Int. J. Res. Mark. 11, 117–125. doi: 10.1016/0167-8116(94)90023-X

[ref3] Arrow K. J. Cropper M. L. Eads G. C. Hahn R. W. Lave L. B. Noll R. G. . (1996). Is there a role for benefit-cost analysis in environmental, health, and safety regulation? Science 272, 221–222. doi: 10.1126/science.272.5259.221, 8602504

[ref4] Attneave F. (1954). Some Informational Aspects of Visual Perception. Psychol. Rev. 61, 183–193. doi: 10.1037/h0054663 13167245

[ref5] Barlow H. B. (1961). Possible principles underlying the transformation of sensory messages. Sensory Communication 1, 217–233.

[ref6] Barnes S. J. (2024). Smooth talking and fast music: understanding the importance of voice and music in travel and tourism ads via acoustic analytics. J. Travel Res. 63, 1070–1085. doi: 10.1177/00472875231185882

[ref7] Barnes S. J. Wang W. (2024). “I like the sound of that”: understanding the effectiveness of audio in ads. Internet Res. 35, 1104–1125. doi: 10.1108/INTR-10-2023-0898

[ref8] Becker G. M. DeGroot M. H. Marschak J. (1964). Measuring utility by a single-response sequential method. Behav. Sci. 9, 226–232. doi: 10.1002/bs.3830090304, 5888778

[ref9] Benjamini Y. Hochberg Y. (1995). Controlling the false discovery rate: a practical and powerful approach to multiple testing. J. R. Stat. Soc. Ser. B Stat Methodol. 57, 289–300. doi: 10.1111/j.2517-6161.1995.tb02031.x

[ref10] Berman M. G. Hout M. C. Kardan O. Hunter M. R. Yourganov G. Henderson J. M. . (2014). The perception of naturalness correlates with Low-level visual features of environmental scenes. PLoS One 9:e114572. doi: 10.1371/journal.pone.0114572, 25531411 PMC4273965

[ref11] Boughanmi K. Ansari A. (2021). Dynamics of musical success: a machine learning approach for multimedia data fusion. J. Mark. Res. 58, 1034–1057. doi: 10.1177/00222437211016495

[ref12] Bougiatiotis K. Giannakopoulos T. (2018). Enhanced movie content similarity based on textual, auditory and visual information. Expert Syst. Appl. 96, 86–102. doi: 10.1016/j.eswa.2017.11.050

[ref13] Breuer C. Rumpf C. (2015). The impact of color and animation on sports viewers’ attention to televised sponsorship signage. J. Sport Manag. 29, 170–183. doi: 10.1123/JSM.2013-0280

[ref14] Chen Y. Nguyen T. V. Kankanhalli M. Yuan J. Yan S. Wang M. (2014). Audio matters in visual attention. IEEE Trans. Circuits Syst. Video Technol. 24, 1992–2003. doi: 10.1109/TCSVT.2014.2329380

[ref15] Choe K. Whittington D. Lauria D. T. (1996). The economic benefits of surface water quality improvements in developing countries: a case study of Davao, Philippines. Land Econ. 72, 519–537. doi: 10.2307/3146913

[ref16] Corrigall K. A. Schellenberg E. G. (2013). “Music: the language of emotion,” in Handbook of Psychology of Emotions (Vol. 2): Recent Theoretical Perspectives and Novel Empirical Findings, 299–325.

[ref17] Coutinho E. Cangelosi A. (2011). Musical emotions: predicting second-by-second subjective feelings of emotion from low-level psychoacoustic features and physiological measurements. Emotion 11, 921–937. doi: 10.1037/a0024700, 21859207

[ref18] Deldjoo Y. Elahi M. Quadrana M. Cremonesi P. (2015). “Toward building a content-based video recommendation system based on Low-level features,” in E-Commerce and Web Technologies, eds. Stuckenschmidt H. Jannach D. (Cham: Springer International Publishing), 45–56.

[ref19] Dong X. Liu H. Xi N. Liao J. Yang Z. (2023). Short video marketing: what, when and how short-branded videos facilitate consumer engagement. Internet Res. 34, 1104–1128. doi: 10.1108/INTR-02-2022-0121

[ref20] Dzyabura D. Peres R. (2024). Color Analytics for Data-Driven Brand Communications. SSRN. Available online at: https://ssrn.com/abstract=5051841 (Accessed January 13, 2025).

[ref21] Einhäuser W. Neubert C. R. Grimm S. Bendixen A. (2024). High visual salience of alert signals can lead to a counterintuitive increase of reaction times. Sci. Rep. 14:8858. doi: 10.1038/s41598-024-58953-4, 38632303 PMC11024089

[ref22] Fong H. Kumar V. Sudhir K. (2025). A theory-based explainable deep learning architecture for music emotion. Mark. Sci. 44, 196–219. doi: 10.1287/mksc.2022.0323, 19642375

[ref23] Foulsham T. Underwood G. (2008). What can saliency models predict about eye movements? Spatial and sequential aspects of fixations during encoding and recognition. J. Vis. 8, 6–6. doi: 10.1167/8.2.6, 18318632

[ref24] Fu S. Cheng X. Bilgihan A. Okumus F. (2024). Hue, brightness, saturation, and caption description: which attributes impact listing preferences on digital accommodation platforms? INTR 34, 495–518. doi: 10.1108/INTR-08-2021-0554

[ref25] Gibson J. (1979). The Ecological Approach to Visual Perception. Boston, MA: Houghton-Miffin Company.

[ref26] Guo P. J. Kim J. Rubin R. (2014). “How video production affects student engagement: an empirical study of MOOC videos,” in Proceedings of the first ACM Conference on Learning @ Scale Conference, (Atlanta Georgia USA: ACM), 41–50.

[ref27] Gurariy G. Randall R. Greenberg A. S. (2021). Manipulation of low-level features modulates grouping strength of auditory objects. Psychol. Res. 85, 2256–2270. doi: 10.1007/s00426-020-01391-4, 32691138

[ref28] Gurariy G. Randall R. Greenberg A. S. (2023). Neuroimaging evidence for the direct role of auditory scene analysis in object perception. Cereb. Cortex 33, 6257–6272. doi: 10.1093/cercor/bhac501, 36562994 PMC10183742

[ref29] Hagtvedt H. Adam Brasel S. (2017). Color saturation increases perceived product size. J. Consum. Res. 44, 396–413. doi: 10.1093/jcr/ucx039

[ref1102] Hasler D. Süsstrunk S. (2003). Measuring colourfulness in natural images., (SPIE), 87–95.

[ref30] Hayes T. R. Henderson J. M. (2021). Deep saliency models learn low-, mid-, and high-level features to predict scene attention. Sci. Rep. 11:18434. doi: 10.1038/s41598-021-97879-z, 34531484 PMC8445969

[ref31] Hollebeek L. D. Glynn M. S. Brodie R. J. (2014). Consumer brand engagement in social media: conceptualization, scale development and validation. J. Interact. Mark. 28, 149–165. doi: 10.1016/j.intmar.2013.12.002

[ref32] Iigaya K. Yi S. Wahle I. A. Tanwisuth K. O’Doherty J. P. (2021). Aesthetic preference for art can be predicted from a mixture of low- and high-level visual features. Nat. Hum. Behav. 5, 743–755. doi: 10.1038/s41562-021-01124-6, 34017097 PMC8494016

[ref33] Itti L. Koch C. (2000). A saliency-based search mechanism for overt and covert shifts of visual attention. Vis. Res. 40, 1489–1506. doi: 10.1016/S0042-6989(99)00163-7, 10788654

[ref34] Itti L. Koch C. (2001). Computational modelling of visual attention. Nat. Rev. Neurosci. 2, 194–203. doi: 10.1038/35058500, 11256080

[ref35] Jalali N. Y. Papatla P. (2016). The palette that stands out: color compositions of online curated visual UGC that attracts higher consumer interaction. Quant. Mark. Econ. 14, 353–384. doi: 10.1007/s11129-016-9178-1

[ref36] Jalil M. Butt F. A. Malik A. (2013) Short-time energy, magnitude, zero crossing rate and autocorrelation measurement for discriminating voiced and unvoiced segments of speech signals., in 2013 The International Conference on Technological Advances in Electrical, Electronics and Computer Engineering (TAEECE) Konya IEEE 208–212.

[ref37] Kalish S. Nelson P. (1991). A comparison of ranking, rating and reservation price measurement in conjoint analysis. Mark. Lett. 2, 327–335. doi: 10.1007/BF00664219

[ref38] Kanuri V. K. Hughes C. Hodges B. T. (2024). Standing out from the crowd: when and why color complexity in social media images increases user engagement. Int. J. Res. Mark. 41, 174–193. doi: 10.1016/j.ijresmar.2023.08.007

[ref39] Kardan O. Demiralp E. Hout M. C. Hunter M. R. Karimi H. Hanayik T. . (2015). Is the preference of natural versus man-made scenes driven by bottom–up processing of the visual features of nature? Front. Psychol. 6:471. doi: 10.3389/fpsyg.2015.00471, 25954228 PMC4407505

[ref40] Kim S. Chung H. (2012). The impacts of perceived fit, brand familiarity, and status consciousness on fashion brand extension evaluation. Int. J. Fash. Des. Technol. Educ. 5, 203–211. doi: 10.1080/17543266.2012.709546

[ref41] Kim J. Gajos K. Shang-Wen L. Miller R. Cai C. (2014). Leveraging Video Interaction Data and Content Analysis to Improve Video Learning. (Toronto, Canada: ACM Press).

[ref42] Klempner L. Epstein A. (2023). The value of video marketing and how you can implement it: strategies for maximizing your presence on YouTube, TikTok, Instagram reels, and Facebook. Orthod. Prod. 30, 20–23.

[ref43] Krajbich I. Armel C. Rangel A. (2010). Visual fixations and the computation and comparison of value in simple choice. Nat. Neurosci. 13, 1292–1298. doi: 10.1038/nn.2635 20835253

[ref44] Krajbich I. Lu D. Camerer C. Rangel A. (2012). The attentional drift-diffusion model extends to simple purchasing decisions. Front. Psychol. 3:193. doi: 10.3389/fpsyg.2012.00193, 22707945 PMC3374478

[ref45] Krishna A. (2012). An integrative review of sensory marketing: engaging the senses to affect perception, judgment and behavior. J. Consum. Psychol. 22, 332–351. doi: 10.1016/j.jcps.2011.08.003

[ref46] Kumar P. (2025) The Rise of Short-Form Video: A Digital Revolution. Int. J. Res. Publ. Rev. doi: 10.5281/ZENODO.15667258

[ref47] Kunz S. Haasova S. Rieß J. Florack A. (2020). Beyond healthiness: the impact of traffic light labels on taste expectations and purchase intentions. Foods 9:134. doi: 10.3390/foods9020134, 32012832 PMC7074546

[ref48] Labrecque L. I. Milne G. R. (2012). Exciting red and competent blue: the importance of color in marketing. J. Acad. Mark. Sci. 40, 711–727. doi: 10.1007/s11747-010-0245-y

[ref49] Le Meur O. Le Callet P. Barba D. (2007). Predicting visual fixations on video based on low-level visual features. Vis. Res. 47, 2483–2498. doi: 10.1016/j.visres.2007.06.015, 17688904

[ref50] Lee S.-H. Liang Y. Chen Y. Mahdi A. Qin J. (2021). Can consumers’ visual attention be predictable? A saliency modelling-based approach on fashion advertisements. Int. J. Fash. Des. Technol. Educ. 14, 253–262. doi: 10.1080/17543266.2021.1925354

[ref51] Lee C.-Y. Tu Y.-N. (2025). Predicting hit songs using audio and visual features. Eng. Proc. 89:43. doi: 10.3390/engproc2025089043

[ref52] Lei S. Wu Q. Du J. (2025). How music tempo influences consumer preferences for advertising with different regulatory focuses: shopping in jumping tempo. J. Advert. Res. 65, 579–596. doi: 10.1080/00218499.2025.2464277, 37339054

[ref53] Li H. Xu J. Fang M. Tang L. Pan Y. (2023). A study and analysis of the relationship between visual—auditory logos and consumer behavior. Behavioral Sciences 13:613. doi: 10.3390/bs13070613, 37504059 PMC10376566

[ref54] Liu X. Shi S. W. Teixeira T. Wedel M. (2018). Video content marketing: the making of clips. J. Mark. 82, 86–101. doi: 10.1509/jm.16.0048

[ref55] Lowe M. L. Haws K. L. (2017). Sounds big: the effects of acoustic pitch on product perceptions. J. Mark. Res. 54, 331–346. doi: 10.1509/jmr.14.0300

[ref1101] Mahapatra D. Winkler S. Yen S.-C. (2008). Motion saliency outweighs other low-level features while watching videos., eds. B. E. Rogowitz and T. N. Pappas (San Jose, CA), 68060P. doi: 10.1117/12.766243

[ref56] Martinez H. Hines A. Farias M. C. Q. (2021). Perceptual quality of audio-visual content with common video and audio degradations. Appl. Sci. 11:5813. doi: 10.3390/app11135813

[ref57] Martin-Gutierrez D. Hernandez Penaloza G. Belmonte-Hernandez A. Alvarez Garcia F. (2020). A multimodal end-to-end deep learning architecture for music popularity prediction. IEEE Access 8, 39361–39374. doi: 10.1109/ACCESS.2020.2976033

[ref58] McFee B. Raffel C. Liang D. Ellis D. McVicar M. Battenberg E. . (2015). Librosa: Audio and Music Signal Analysis in Python. (Austin, Texas: SciPy), 18–24. doi: 10.25080/Majora-7b98e3ed-003

[ref59] Milosavljevic M. Navalpakkam V. Koch C. Rangel A. (2012). Relative visual saliency differences induce sizable bias in consumer choice. J. Consum. Psychol. 22, 67–74. doi: 10.1016/j.jcps.2011.10.002

[ref60] Mindak W. A. (1956). A new technique for measuring advertising effectiveness. J. Mark. 20, 367–378. doi: 10.2307/1248238

[ref61] Mital P. K. Smith T. J. Hill R. L. Henderson J. M. (2011). Clustering of gaze during dynamic scene viewing is predicted by motion. Cogn. Comput. 3, 5–24. doi: 10.1007/s12559-010-9074-z

[ref62] Opoku-Baah C. Schoenhaut A. M. Vassall S. G. Tovar D. A. Ramachandran R. Wallace M. T. (2021). Visual influences on auditory behavioral, neural, and perceptual processes: a review. JARO 22, 365–386. doi: 10.1007/s10162-021-00789-0, 34014416 PMC8329114

[ref63] Oren S. Sela T. Levy D. J. Schonberg T. (2020). Item features interact with item category in their influence on preferences. Front. Psychol. 11:988. doi: 10.3389/fpsyg.2020.00988, 32793016 PMC7391002

[ref64] Ostensson O. (2001). Valuing environmental preferences: theory and practice of the contingent valuation method in the US, EU and developing countries: Ian J. Bateman and Kenneth G. Willis (Eds.); Oxford university press, New York, 1999, 645 pp., ISBN 0-19-828853-0 (hardcover). Resour. Policy 27, 57–59. doi: 10.1016/S0301-4207(00)00045-3

[ref65] Overgoor G. Rand W. Van Dolen W. Mazloom M. (2022). Simplicity is not key: understanding firm-generated social media images and consumer liking. Int. J. Res. Mark. 39, 639–655. doi: 10.1016/j.ijresmar.2021.12.005

[ref66] Pazda A. D. Thorstenson C. A. Fetterman A. K. (2024). Colorfulness influences perceptions of valence and arousal. J. Exp. Psychol. Gen. 153, 145–158. doi: 10.1037/xge0001484, 37870815

[ref67] Pech-Pacheco J. L. Cristóbal G. Chamorro-Martínez J. Fernández-Valdivia J. (2000). “Diatom autofocusing in brightfield microscopy: a comparative study,” in Proceedings 15th International Conference on Pattern Recognition ICPR-2000, 3, 314–317. doi: 10.1109/ICPR.2000.903548

[ref68] Peterson R. A. Cannito M. P. Brown S. P. (1995). An exploratory investigation of voice characteristics and selling effectiveness. J. Pers. Sell. Sales Manag. 15, 1–15. doi: 10.1080/08853134.1995.10754008

[ref69] Petty R. E. Cacioppo J. T. (1986). “The elaboration likelihood model of persuasion,” in Advances in Experimental Social Psychology, ed. Berkowitz L. (Academic Press), 123–205. doi: 10.1016/S0065-2601(08)60214-2

[ref70] Pieters R. Wedel M. Batra R. (2010). The stopping power of advertising: measures and effects of visual complexity. J. Mark. 74, 48–60. doi: 10.1509/jmkg.74.5.48

[ref71] Pons J. Lidy T. Serra X. (2016). “Experimenting with musically motivated convolutional neural networks,” in 2016 14th International Workshop on Content-Based Multimedia Indexing (CBMI), (Bucharest, Romania: IEEE), 1–6.

[ref72] Robinson C. W. Chandra M. Sinnett S. (2016). Existence of competing modality dominances. Atten. Percept. Psychophys. 78, 1104–1114. doi: 10.3758/s13414-016-1061-3, 26832916

[ref73] Rumpf C. Boronczyk F. Breuer C. (2020). Predicting consumer gaze hits: a simulation model of visual attention to dynamic marketing stimuli. J. Bus. Res. 111, 208–217. doi: 10.1016/j.jbusres.2019.03.034

[ref74] Sample K. L. Hagtvedt H. Brasel S. A. (2020). Components of visual perception in marketing contexts: a conceptual framework and review. J. Acad. Mark. Sci. 48, 405–421. doi: 10.1007/s11747-019-00684-4

[ref75] Schwenzow J. Hartmann J. Schikowsky A. Heitmann M. (2021). Understanding videos at scale: how to extract insights for business research. J. Bus. Res. 123, 367–379. doi: 10.1016/j.jbusres.2020.09.059

[ref76] Shams L. Kamitani Y. Shimojo S. (2000). What you see is what you hear. Nature 408, 788–788. doi: 10.1038/35048669, 11130706

[ref77] Shannon C. E. (1948). A mathematical theory of communication. Bell Syst. Tech. J. 27, 379–423. doi: 10.1002/j.1538-7305.1948.tb01338.x

[ref78] Shi A. Huo F. Hou G. (2021). Effects of design aesthetics on the perceived value of a product. Front. Psychol. 12:670800. doi: 10.3389/fpsyg.2021.670800, 34393904 PMC8359925

[ref79] Shin D. He S. Lee G. M. Whinston A. B. Cetintas S. Lee K.-C. (2020). Enhancing social media analysis with visual data analytics: a deep learning approach. MIS Q. 44, 1459–1492. doi: 10.25300/MISQ/2020/14870

[ref80] Shou Z. Pan J. Chan J. Miyazawa K. Mansour H. Vetro A. . (2018). Online Detection of Action Start in Untrimmed, Streaming Videos, in European Conference on Computer Vision, 534–551.

[ref81] Simmonds L. Bogomolova S. Kennedy R. Nenycz-Thiel M. Bellman S. (2020). A dual-process model of how incorporating audio-visual sensory cues in video advertising promotes active attention. Psychol. Mark. 37, 1057–1067. doi: 10.1002/mar.21357

[ref82] Simola J. Kuisma J. Kaakinen J. K. (2020). Attention, memory and preference for direct and indirect print advertisements. J. Bus. Res. 111, 249–261. doi: 10.1016/j.jbusres.2019.06.028

[ref83] Spence C. (2009). “Explaining the Colavita visual dominance effect,” in Progress in Brain Research, (Elsevier), 245–258. doi: 10.1016/S0079-6123(09)17615-X 19733761

[ref84] Sunaga T. (2018). How the sound frequency of background music influences consumers’ perceptions and decision making. Psychol. Mark. 35, 253–267. doi: 10.1002/mar.21084

[ref85] Tavakoli H. R. Borji A. Rahtu E. Kannala J. (2020). DAVE: A Deep Audio-Visual Embedding for Dynamic Saliency Prediction. *arXiv [Preprint]*. doi: 10.48550/arXiv.1905.10693

[ref86] Tavares G. Perona P. Rangel A. (2017). The attentional drift diffusion model of simple perceptual decision-making. Front. Neurosci. 11:468. doi: 10.3389/fnins.2017.00468, 28894413 PMC5573732

[ref87] Towal R. B. Mormann M. Koch C. (2013). Simultaneous modeling of visual saliency and value computation improves predictions of economic choice. Proc. Natl. Acad. Sci. USA 110, E3858–E3867. doi: 10.1073/pnas.1304429110, 24019496 PMC3791693

[ref88] Trunfio M. Rossi S. (2021). Conceptualising and measuring social media engagement: a systematic literature review. Ital. J. Mark. 2021, 267–292. doi: 10.1007/s43039-021-00035-8

[ref89] Van Paasschen J. Zamboni E. Bacci F. Melcher D. (2014). Consistent emotions elicited by low-level visual features in abstract art. Art Percept. 2, 99–118. doi: 10.1163/22134913-00002012

[ref90] Velardo V. (2020) AudioSignalProcessingForML. Availble online at: https://github.com/musikalkemist/AudioSignalProcessingForML/tree/2b7efd37305a10d0059fdd0a41f011fd0218771a/9-%20RMS%20energy%20and%20zero-crossing%20rate (Accessed December 11, 2024).

[ref91] Vroomen J. de Gelder B. (2000). Sound enhances visual perception: cross-modal effects of auditory organization on vision. J. Exp. Psychol. Hum. Percept. Perform. 26, 1583–1590. doi: 10.1037/0096-1523.26.5.1583, 11039486

[ref92] Wertenbroch K. Skiera B. (2002). Measuring consumers’ willingness to pay at the point of purchase. J. Mark. Res. 39, 228–241. doi: 10.1509/jmkr.39.2.228.19086

[ref93] Williams J. R. Markov Y. A. Tiurina N. A. Störmer V. S. (2022). What you see is what you hear: sounds Alter the contents of visual perception. Psychol. Sci. 33, 2109–2122. doi: 10.1177/09567976221121348, 36179072

[ref94] Wong L.-K. Low K.-L. (2011). “Saliency retargeting: an approach to enhance image aesthetics,” in 2011 IEEE Workshop on Applications of Computer Vision (WACV), (Kona, HI: IEEE), 73–80.

[ref95] Xiao L. Li X. Mou J. (2024). Exploring user engagement behavior with short-form video advertising on short-form video platforms: a visual-audio perspective. Internet Res. 36, 154–188. doi: 10.1108/INTR-07-2023-0521

[ref96] Yang Q. Wang Y. Song M. Jiang Y. Li Q. (2025). Sonic strategies: unveiling the impact of sound features in short video ads on enterprise market entry performance. J. Bus. Bus. Mark. 32, 95–116. doi: 10.1080/1051712X.2024.2396966

[ref97] Yang J. Xie Y. Krishnamurthi L. Papatla P. (2022). High-energy ad content: a large-scale investigation of TV commercials. J. Mark. Res. 59, 840–859. doi: 10.1177/00222437211067802

[ref98] Yang J. Zhang J. Zhang Y. (2025). Engagement that sells: influencer video advertising on TikTok. Mark. Sci. 44, 247–267. doi: 10.1287/mksc.2021.0107

[ref99] Yorkston E. Menon G. (2004). A sound idea: phonetic effects of brand names on consumer judgments. J. Consum. Res. 31, 43–51. doi: 10.1086/383422

[ref100] Zangerle E. Vötter M. Huber R. Yang Y.-H. (2019) Hit Song Prediction: Leveraging Low- and High-Level Audio Features., in *ISMIR*, 319–326.

[ref101] Zhang Z. Qiu K. Ye Y. (2025). Influence of audiovisual features of short video advertising on consumer engagement behaviors: evidence from TikTok. J. Bus. Res. 201:115662. doi: 10.1016/j.jbusres.2025.115662

[ref102] Zhou M. Chen G. H. Ferreira P. Smith M. D. (2021). Consumer behavior in the online classroom: using video analytics and machine learning to understand the consumption of video courseware. J. Mark. Res. 58, 1079–1100. doi: 10.1177/00222437211042013

