# Supplementary material for: Low-level visual and auditory features predict preferences of commercials
Source: Front Psychol. 2026 Oct 29;17:1918605. doi: 10.3389/fpsyg.2026.1918605 (PMC13754823; doi:10.3389/fpsyg.2026.1918605)
Supplement: Supplementary file 1 [file Data_Sheet_1.PDF]

Low Level Visual and Auditory Features Predict Preferences of Commercials

Appendix

A1. List of Commercials Used in the Study

- *Table A1. Commercial Stimuli (Item Index, ID, and YouTube Links)*

A2. Effect of Age on Subjective Values (Liking and WTP)

A3. Visual Feature Selection and Elimination Steps

- *Table A2. Specific Visual Features Eliminated and Retained Across Selection Steps*

A4. Visual Feature Correlation Matrix

- *Figure A1. Pearson Correlation Matrix of Low-Level Visual Features*

A5. Auditory Feature Selection and Elimination Steps

- *Table A3. Specific Auditory Features Eliminated and Retained Across Selection Steps*

A6. Auditory Feature Correlation Matrix

- *Figure A2. Pearson Correlation Matrix of Low-Level Auditory Features*

A7. Ridge Regression Results for Visual Features Predicting WTP

- *Table A4. Out-of-Sample Feature Coefficients for Visual Model (WTP)*

A8. Ridge Regression Results for Visual Features Predicting Liking

- *Table A5. Out-of-Sample Feature Coefficients for Visual Model (Liking)*

A9. Ridge Regression Results for Auditory Features Predicting WTP

- *Table A6. Out-of-Sample Feature Coefficients for Auditory Model (WTP)*

A10. Ridge Regression Results for Auditory Features Predicting Liking

- *Table A7. Out-of-Sample Feature Coefficients for Combined Model (Liking)*

A11. Ridge Regression Results for Combined Model Predicting WTP

- *Table A8. Out-of-Sample Feature Coefficients for Combined Model (WTP)*

A12. Ridge Regression Results for Combined Model Predicting Liking

- *Table A9. Out-of-Sample Feature Coefficients for Combined Model (Liking)*

A13. Visual and Auditory Feature Correlation Matrix

- *Figure A3. Pearson Correlation Matrix of Low-Level Visual and Auditory Features*

A14. Main analysis without feature selection

- *Fig A4. Prediction results for low-level visual features*
- *Fig A5. Low-level visual influential features*

A15. Kernel Counts for the SVR Models

- *Table A10. Kernel Selection Frequency across Feature Sets and Targets*

A16. Classification Task: Binary Analysis

- *Fig A6. Mean Accuracy Scores for Original and Shuffled Data*

A17. Performance Metrics of Predictive Models

- *Table A11. Performance Metrics for Predicting Liking (Accuracy, Sensitivity, Specificity, Precision, NPV)*
- *Table A12. Performance Metrics for Predicting WTP (Accuracy, Sensitivity, Specificity, Precision, NPV)*

A1. List of Commercials Used in the Study

Table A1 presents all commercials included in the analysis, along with their item index, internal ID, and YouTube link.

Table A1. Commercial Stimuli

Item Index	ID	Link
1001	Quaker_1	https://www.youtube.com/watch?v=vLNQH3epcw0
1004	Quaker_4	https://www.youtube.com/watch?v=8B7-XywGfDU
1005	Quaker_5	https://www.youtube.com/watch?v=_QW7WrPPTAI
1006	Quaker_6	https://www.youtube.com/watch?v=hnk7nyM7vNw
2004	FuzeTea_4	https://www.youtube.com/watch?v=fCcfkyoh4F4
2005	FuzeTea_5	https://www.youtube.com/watch?v=VyFPWEfcL2U
2006	FuzeTea_6	https://www.youtube.com/watch?v=2yGC8wytHEg
3001	Filtuna_1	https://www.youtube.com/watch?v=4N60iyLH1gg
3002	Filtuna_2	https://www.youtube.com/watch?v=O5nX7YRbrVM
3003	Filtuna_3	https://www.youtube.com/watch?v=yUJN0Nc1MrQ
3004	Filtuna_4	https://www.youtube.com/watch?v=1DEyH6UeBCw&list=UUi3_u19KpyhZC6XxFhL41oA&index=6
3006	Filtuna_6	https://www.youtube.com/watch?v=wfddqohpQoA&list=UUi3_u19KpyhZC6XxFhL41oA&index=11
4001	GelDeLin_1	https://www.youtube.com/watch?v=wbeJR41Rd7Q
4002	GelDeLin_2	https://www.youtube.com/watch?v=ibur4fRWgDA
4003	GelDeLin_3	https://www.youtube.com/watch?v=Cb0hziJetYA
5003	Baracke_3	https://www.youtube.com/watch?v=vn8rQR609Y0
5004	Baracke_4	https://www.youtube.com/watch?v=ekAxg2qmA7U
5005	Baracke_5	https://www.youtube.com/watch?v=LZ4pzw6KWBg
5006	Baracke_6	https://www.youtube.com/watch?v=oY0a3n6M29w
5011	Oreo_1	https://www.youtube.com/watch?v=O7G8MejfBYM
5012	Oreo_2	https://www.youtube.com/watch?v=52JG4OuAsUM
5013	Oreo_3	https://www.youtube.com/watch?v=DF4ViwhKc00
3011	YadMordechai_1	https://www.youtube.com/watch?v=mqKxve0OzHA
3012	YadMordechai_2	https://www.youtube.com/watch?v=VQk537vN64o
3013	YadMordechai_3	https://www.youtube.com/watch?v=s4JG08e_TKE
3021	Kikkoman_1	https://www.youtube.com/watch?v=gNoMNotRvmc
3022	Kikkoman_2	https://www.youtube.com/watch?v=sbhxOMQ159k
3024	Kikkoman_4	https://www.youtube.com/watch?v=Lu-b_5rzr_E
3026	Kikkoman_6	https://www.youtube.com/watch?v=YECe3eak-wQ
3027	Kikkoman_7	https://www.youtube.com/watch?v=fy3IwTV6Glw
3031	Prince_1	https://www.youtube.com/watch?v=1OwIQ0yTZts
3032	Prince_2	https://www.youtube.com/watch?v=jLlbr2xRAHo
3033	Prince_3	https://www.youtube.com/watch?v=jdI3vlp1sOY
3034	Prince_4	https://www.youtube.com/watch?v=bYV9YsdMZGo
3041	HEINZ_1	https://www.youtube.com/watch?v=g349B30kXmY
3042	HEINZ_2	https://www.youtube.com/watch?v=9UnU_k0628s
3043	HEINZ_3	https://www.youtube.com/watch?v=3UZyOeX71ho
3044	HEINZ_4	https://www.youtube.com/watch?v=8K3J_vWIDL8
3045	HEINZ_5	https://www.youtube.com/watch?v=wJTcY3-al-s
1011	Branflakes_1	https://www.youtube.com/watch?v=BCnBi0wyvQs
1012	Branflakes_2	https://www.youtube.com/watch?v=ohSYDOfvmvw
1013	Branflakes_3	https://www.youtube.com/watch?v=AfsUsB0V-hA

1014	Branflakes_4	https://www.youtube.com/watch?v=u9Pr6aK2154
1021	Branflakesextra_1	https://www.youtube.com/watch?v=leY2XlSPeHw
1022	Branflakesextra_2	https://www.youtube.com/watch?v=IYrCDaIvGyM
1023	Branflakesextra_3	https://www.youtube.com/watch?v=36T8mbVijLY
1024	Branflakesextra_4	https://www.youtube.com/watch?v=RBDGuKxjYtA
1025	Branflakesextra_5	https://www.youtube.com/watch?v=dNTm0bJihog
5021	Tapuchips_1	https://www.youtube.com/watch?v=zVBKq9iulvk
5022	Tapuchips_2	https://www.youtube.com/watch?v=N3olqlyG_pk
5023	Tapuchips_3	https://www.youtube.com/watch?v=h7Srb129KMQ
5024	Tapuchips_4	https://www.youtube.com/watch?v=cvplaWREZEs
5031	Parachocolate_1	https://www.youtube.com/watch?v=-pUXaqpxMos
5032	Parachocolate_2	https://www.youtube.com/watch?v=DieP8Khzn4U
5033	Parachocolate_3	https://www.youtube.com/watch?v=59aPrHPkokc
5041	Splendid_1	https://www.youtube.com/watch?v=FpNTBXLczXg&list=RDCMUCofCmUoYZWF_WUxn6qdST4w&start_radio=1&t=3
5042	Splendid_2	https://www.youtube.com/watch?v=0bQsOCDSU4s
5043	Splendid_3	https://www.youtube.com/watch?v=Qy4nxnsOVbM
5044	Splendid_4	https://www.youtube.com/watch?v=3n3zx1rmOy8
5051	Fitness_1	https://www.youtube.com/watch?v=CVcDAoxD48o
5052	Fitness_2	https://www.youtube.com/watch?v=0SjbnjBLfqo
5053	Fitness_3	https://www.youtube.com/watch?v=xZknGB8d9kA
5054	Fitness_4	https://www.youtube.com/watch?v=5qnzgeGfiCo
5055	Fitness_5	https://www.youtube.com/watch?v=ajyeNv_X6Kk
2011	Cafeelite_1	https://www.youtube.com/watch?v=R53vojvOGCE
2012	Cafeelite_2	https://www.youtube.com/watch?v=eSsOnR5k-ac
2013	Cafeelite_3	https://www.youtube.com/watch?v=UDtC70NJLwo
2021	Chocolatemilk_1	https://www.youtube.com/watch?v=wDlPcpAjUFg
2022	Chocolatemilk_2	https://www.youtube.com/watch?v=illqhwMNf0k
2023	Chocolatemilk_3	https://www.youtube.com/watch?v=8oJbfY-8oe8
2031	Wissotzky_1	https://www.youtube.com/watch?v=SHnl5ZLMh18
2032	Wissotzky_2	https://www.youtube.com/watch?v=KLDoE2g6QjE
2034	Wissotzky_4	https://www.youtube.com/watch?v=pq23TPxHG0E
2035	Wissotzky_5	https://www.youtube.com/watch?v=0_CNrxepbZI
2036	Wissotzky_6	https://www.youtube.com/watch?v=89CWvslyOHk
2041	CocaCola2L_1	https://www.youtube.com/watch?v=RNKejbYQhOI
2042	CocaCola2L_2	https://www.youtube.com/watch?v=dG1UNaQ5hXg
2043	CocaCola2L_3	https://www.youtube.com/watch?v=LMcEQv7JorY
2051	Neviot_1	https://www.youtube.com/watch?v=zwkxRBmpyOI
2052	Neviot_2	https://www.youtube.com/watch?v=E3hW9c3xAZE
2053	Neviot_3	https://www.youtube.com/watch?v=uWYcSkWtPuw
2054	Neviot_4	https://www.youtube.com/watch?v=re6ECEb8gg8
2056	Neviot_6	https://www.youtube.com/watch?v=z6T24hXmhmM
4011	Colgate_1	https://www.youtube.com/watch?v=Y5oVf1Lpwxk
4012	Colgate_2	https://www.youtube.com/watch?v=Gd2WziS3kqI
4013	Colgate_3	https://www.youtube.com/watch?v=aMA4IrMtNxA
4015	Colgate_5	https://www.youtube.com/watch?v=sHc2I2GWpuU
4016	Colgate_6	https://www.youtube.com/watch?v=p-54QoI3P5U
4021	Zippersmallbag_1	https://www.youtube.com/watch?v=9-PCcy0tZuw
4022	Zippersmallbag_2	https://www.youtube.com/watch?v=7G-DPFCLGRQ
4023	Zippersmallbag_3	https://www.youtube.com/watch?v=W1oi6w1RcdQ
4031	Zipperbigbag_1	https://www.youtube.com/watch?v=A_XqgYVUsYI
4032	Zipperbigbag_2	https://www.youtube.com/watch?v=aBJbIMPAZI8
4033	Zipperbigbag_3	https://www.youtube.com/watch?v=grjVvUzGg0Q
4041	Sano99_1	https://www.youtube.com/watch?v=TM1QJuoyRIw
4042	Sano99_2	https://www.youtube.com/watch?v=iBqAit6REok
4043	Sano99_3	https://www.youtube.com/watch?v=gf-aLZ7dVKA
1031	ChocolateSpread_1	https://www.youtube.com/watch?v=ztGuaWEbHgA
1032	ChocolateSpread_2	https://www.youtube.com/watch?v=5bJSyZKC2Z4
1033	ChocolateSpread_3	https://www.youtube.com/watch?v=LK9XirwvfAg

A2. Effect of Age subjective value

To evaluate whether participant age influenced evaluations, we fitted linear mixed-effects regression models with random intercepts per participant.

$$Y_{ij} = \beta_0 + \beta_1 * Age_i + \mu_i + \epsilon_{ij}$$

Where Y_{ij} is either Liking or WTP for participant i in commercial j. β_0 is the fixed intercept, and β_1 is the fixed-effect slope coefficient for the Age_i. μ_i represents the Random intercept for participant i , and ϵ_{ij} represents the residual error.

The effect of age on liking was not statistically significant (β= 0.016, SE = 0.010, z = 1.66, p = 0.097). This indicates that liking for the commercials was consistent across age groups.

The effect of age on WTP, on the other hand, was statistically significant, showing a positive association (β = 0.377, SE = 0.168, z = 2.25, p = 0.025). On average, each additional year of age was associated with an increase of approximately 0.38 tokens in WTP ratings.

A3. Specific visual features eliminated and retained

Table A2 documents which visual features were retained and eliminated in every step of the feature selection.

Table A2. Specific Visual Features Eliminated and Retained Across Selection Steps

Exclusion Step	Reason for Exclusion	Features eliminated	Features keep
Step 1	We narrowed the initial 109 features down to 48 video-level features, prioritizing aggregate video-level metrics over individual frame-level metrics	Person, Bicycle, Car, Motorcycle, Airplane, Bus, Train, Truck, Boat, Traffic light, Fire hydrant, Stop sign, Parking meter, Bench, Bird, Cat, Dog, Horse, Sheep, Cow, Elephant, Bear, Zebra, Giraffe, Backpack, Umbrella, Handbag, Tie, Suitcase, Frisbee, Skis, Snowboard, Sports ball, Kite, Baseball bat, Baseball glove, Skateboard, Surfboard, Tennis racquet, Bottle, Cake, Chair, Couch, Potted plant, Bed, Dining table, Toilet, TV, Laptop, Mouse, Remote, Keyboard, Cell phone, Microwave, Oven, Toaster, Sink, Refrigerator, Book, Clock, Vase, Scissors, Wine glass, Cup, Fork, Knife, Spoon, Bowl, Banana, Apple, Sandwich, Orange (fruit), Broccoli, Carrot, Hot dog, Pizza, Donut, Teddy	Average Colorfulness, Variance of Colorfulness, Average Saturation, Variance of Saturation, Average Luminosity, Variance of Luminosity, Average Black, Variance of Black, Average Blue, Variance of Blue , Average Brown, Variance of Brown, Average Gray, Variance of Gray, Average Green, Variance of Green, Average Orange, Variance of Orange, Average Pink, Variance of Pink, Average Purple, Variance of Purple, Average Red, Variance of Red, Average White, Variance of White, Average Yellow, Variance of Yellow, Average Anger, Maximum Anger, Average Disgust, Maximum Disgust, Average Fear, Maximum Fear, Average Happiness, Maximum

		bear, Hair drier, Toothbrush, Colorfulness, Saturation, Luminosity, Black, Blue, Brown, Gray, Green, Orange, Pink, Purple, Red, White, Yellow, Anger, Disgust, Fear, Happiness, Sadness, Surprise, Neutral, Face Confidence & Bounding Box, Object confidence score, Frame quality, ResNet-152 visual embedding, Scene start/end frame	Happiness, Average Sadness, Maximum Sadness, Average Surprise, Maximum Surprise, Average Neutral, Maximum Neutral, Average Number of Faces, Share of Frames with Faces (Face Ratio), Human Area Coverage, Average Quality, Average Scene Cut Frequency, , Video Length
Step 2	High missingness (>50% missing values)	Average Anger, Maximum Anger, Average Disgust, Maximum Disgust, Average Fear, Maximum Fear, Average Happiness, Maximum Happiness, Average Sadness, Maximum Sadness, Average Surprise, Maximum Surprise, Average Neutral, Maximum Neutral	Average Colorfulness, Variance of Colorfulness, Average Saturation, Variance of Saturation, Average Luminosity, Variance of Luminosity, Average Black, Variance of Black, Average Blue, Variance of Blue , Average Brown, Variance of Brown, Average Gray, Variance of Gray, Average Green, Variance of Green, Average Orange, Variance of Orange, Average Pink, Variance of Pink, Average Purple, Variance of Purple, Average Red, Variance of Red, Average White, Variance of White, Average Yellow, Variance of Yellow, Average Number of Faces, Share of Frames with Faces (Face Ratio), Human Area Coverage, Average Quality, Average Scene Cut Frequency
Step 3	Specific color channel (retained overall colorfulness)	Variance of Black, Average Blue, Variance of Blue , Average Brown, Variance of Brown, Average Gray, Variance of Gray, Average Green, Variance of Green, Average Orange, Variance of Orange, Average Pink, Variance of Pink, Average Purple, Variance of Purple, Average Red, Variance of Red, Average White, Variance of White, Average Yellow, Variance of Yellow	Average Colorfulness, Variance of Colorfulness, Average Saturation, Variance of Saturation, Average Luminosity, Variance of Luminosity, Average Number of Faces, Share of Frames with Faces (Face Ratio), Human Area Coverage, Average Quality, Average Scene Cut Frequency
Step 4	Multicollinearity	Average Number of Faces	Average Colorfulness, Variance of Colorfulness, Average Saturation, Variance of Saturation, Average Luminosity, Variance of Luminosity, Share of Frames with Faces (Face Ratio), Human Area Coverage, Average Quality, Average Scene Cut Frequency

A4. Correlation of visual features and elimination for multicollinearity

Figure A1 shows a heat map of the correlation matrix used to select visual features in the process of eliminating features due to multicollinearity during feature selection.

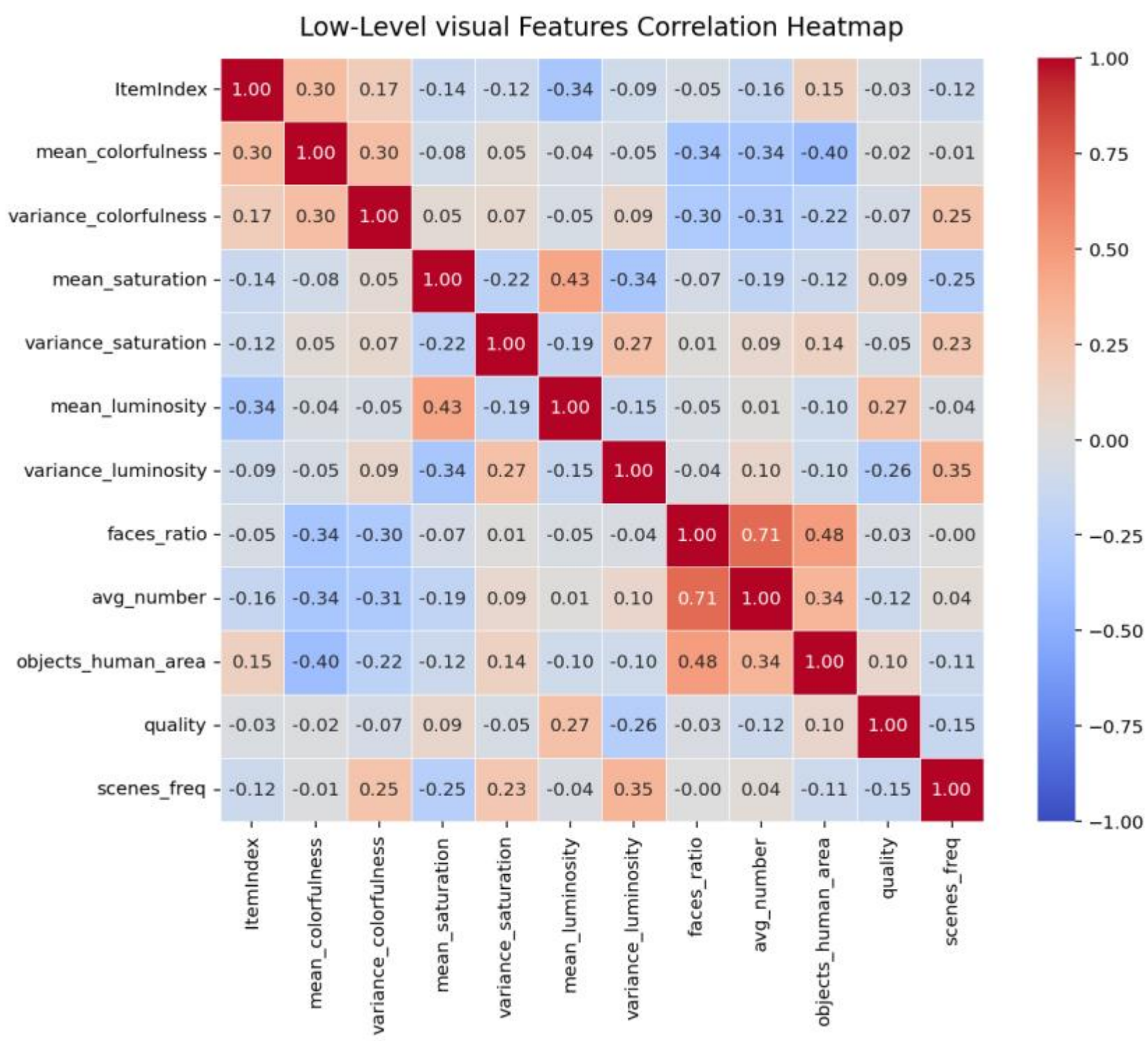

Figure A1. *Pearson correlation matrix of low-level visual features.*

Note. Heatmap displaying pairwise Pearson correlation coefficients across extracted low-level visual features for the commercial stimuli (N = 100). Values range from -1.00 (strong negative correlation) to +1.00 (strong positive correlation), as indicated by the color spectrum (coolwarm). Numerical annotations within each cell represent rounded correlation coefficients. Correlation matrices were computed to assess feature redundancy and inspect multicollinearity (r > 0.70) prior to feature selection and predictive modeling.

A5. Specific auditory features eliminated and retained

Table A3 documents which auditory features were retained and eliminated at each step of feature selection.

Table A3. Specific Auditory Features Eliminated and Retained Across Selection Steps

Exclusion Step	Reason for Exclusion	Features eliminated	Features keep
Step 1	Multicollinearity	Spectral Centroid mean, Spectral Centroid variance, Band Energy Ratio variance	Root mean-square energy mean, Root mean-square energy variance, zero crossing rate mean, zero crossing rate variance, tempo mean, tempo variance, Spectral Bandwidth mean, Spectral Bandwidth variance, Band Energy Ratio mean

A6. Correlation of auditory features and elimination for multicollinearity

Figure A2 shows a heat map of the correlation matrix used to select auditory features in the process of eliminating features due to multicollinearity during feature selection.

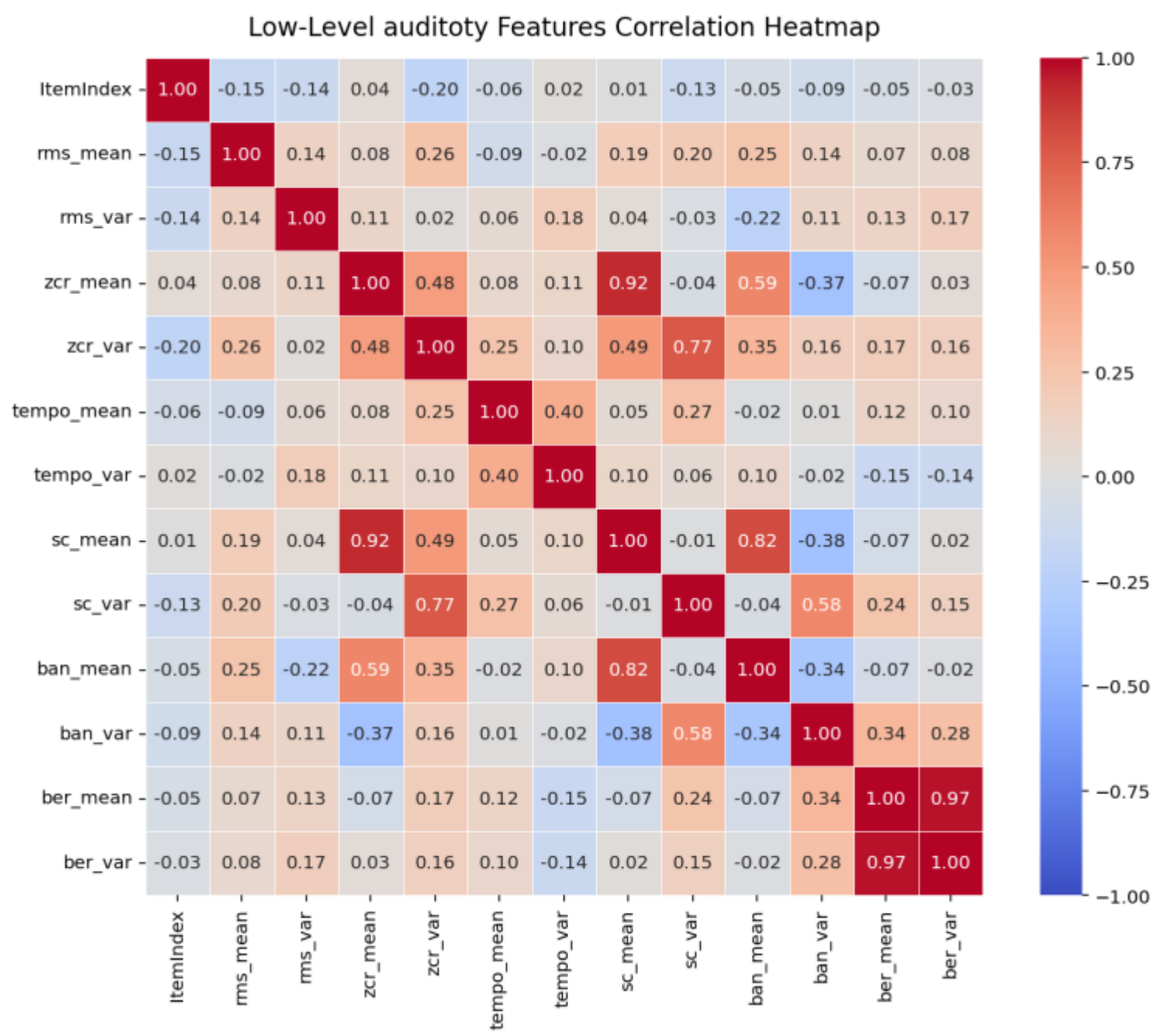

Figure A2. *Pearson correlation matrix of low-level auditory features.*

Note. Heatmap displaying pairwise Pearson correlation coefficients across extracted low-level auditory features for the commercial stimuli (N = 100). Values range from -1.00 (strong negative correlation) to +1.00 (strong positive correlation), as indicated by the color spectrum (coolwarm). Numerical annotations within each cell represent rounded correlation coefficients. Correlation matrices were computed to assess feature redundancy and inspect multicollinearity (r > 0.70) prior to feature selection and predictive modeling.

A7. Ridge Regression Results for Visual Features Predicting WTP

Reports average feature weights, raw p-values, and FDR-corrected p-values for the visual-only linear ridge regression predicting Willingness-To-Pay (WTP).

Table A4. Out-of-Sample Feature Coefficients for Visual Model (WTP)

Feature	Average Coefficient	P value	Corrected P-Value
mean colorfulness	-0.00859164	0.9354	0.935400
variance colorfulness	-0.244091	0.0130	0.065000
mean saturation	-0.102841	0.3244	0.648800
variance saturation	0.0401882	0.7026	0.885000
mean luminosity	0.156767	0.1184	0.394667
variance luminosity	-0.141437	0.1694	0.423500
faces ratio	-0.0523503	0.6130	0.885000
Objects human area	0.0216021	0.8338	0.926444
Quality	0.0355283	0.7080	0.885000
Scene frequency	0.45298	0.0000	0.000000

Note: Coefficients (β) represent average feature weights averaged across all N=100 Leave-One-Out (LOO) cross-validation iterations using standardized features. p-values were computed across iterations, and p-value adjustments were calculated using Benjamini–Hochberg False Discovery Rate (FDR) correction (α=.05) across the 10 visual features.

A8. Ridge Regression Results for Visual Features Predicting Liking

Reports average feature weights, raw p-values, and FDR-corrected p-values for the visual-only linear ridge regression predicting Liking ratings.

Table A5. Out-of-Sample Feature Coefficients for Visual Model (Liking)

Feature	Average Coefficient	P value	Corrected P-Value
mean colorfulness	-0.034611	0.7346	0.734600
variance colorfulness	-0.206031	0.0328	0.082000
mean saturation	-0.319166	0.0014	0.004667
variance saturation	0.144132	0.1410	0.176250
mean luminosity	0.312336	0.0014	0.004667
variance luminosity	-0.159920	0.1154	0.164857
faces ratio	-0.179926	0.0796	0.154000
Objects human area	0.140252	0.1864	0.207111
Quality	-0.158736	0.0924	0.154000
Scene frequency	0.367917	0.0000	0.000000

Note: Coefficients (β) represent average feature weights averaged across all N=100 Leave-One-Out (LOO) cross-validation iterations using standardized features. p-values were computed across iterations, and p-value adjustments were calculated using Benjamini–Hochberg False Discovery Rate (FDR) correction (α=.05) across the 10 visual features.

A9. Ridge Regression Results for Auditory Features Predicting WTP

Reports average feature weights, raw p-values, and FDR-corrected p-values for the auditory-only linear ridge regression predicting Willingness-To-Pay (WTP).

Table A6. Out-of-Sample Feature Coefficients for Auditory Model (WTP)

Feature	Average Coefficient	P value	Corrected P-Value
ber_mean	0.002003	0.9822	0.982200
rms_mean	-0.046233	0.6426	0.826200
rms_var	-0.102428	0.2878	0.647550
zcr_mean	-0.371648	0.0020	0.009000
zcr_var	0.438840	0.0000	0.000000
tempo_mean	0.225751	0.0192	0.057600
tempo_var	0.055645	0.5756	0.826200
ban_mean	-0.041110	0.7350	0.826875
ban_var	0.073434	0.4976	0.826200

Note: Coefficients (β) represent average feature weights averaged across all N=100 Leave-One-Out (LOO) cross-validation iterations using standardized features. p-values were computed across iterations, and p-value adjustments were calculated using Benjamini–Hochberg False Discovery Rate (FDR) correction (α=.05) across the 9 auditory features (ber: Band Energy Ratio; rms: Root Mean Square energy; zcr: Zero Crossing Rate; ban: Spectral Bandwidth).

A10. Ridge Regression Results for Auditory Features Predicting Liking

Reports average feature weights, raw p-values, and FDR-corrected p-values for the auditory-only linear ridge regression predicting Liking ratings.

Table A7. Out-of-Sample Feature Coefficients for Auditory Model (Liking)

Feature	Average Coefficient	P value	Corrected P-Value
ber_mean	-0.038106	.7212	0.7634

rms_mean	0.056391	0.5686	0.7634
rms_var	0.068817	0.4756	0.7634
zcr_mean	-0.398338	0.0006	0.0027
zcr_var	0.405138	0.0002	0.0018
tempo_mean	0.044717	0.6476	0.7634
tempo_var	-0.030523	0.7634	0.7634
ban_mean	-0.119120	0.2972	0.6687
ban_var	-0.176892	0.0980	0.2940

Note: Coefficients (β) represent average feature weights averaged across all N=100 Leave-One-Out (LOO) cross-validation iterations using standardized features. p-values were computed across iterations, and p-value adjustments were calculated using Benjamini–Hochberg False Discovery Rate (FDR) correction (α=.05) across the 9 auditory features (ber: Band Energy Ratio; rms: Root Mean Square energy; zcr: Zero Crossing Rate; ban: Spectral Bandwidth).

A11. Ridge Regression Results for Combined Model Predicting WTP

Reports average feature weights, raw p-values, and FDR-corrected p-values for the joint visual and auditory linear ridge regression predicting Willingness-To-Pay (WTP).

Table A8. Out-of-Sample Feature Coefficients for Combined Model (WTP)

Feature	Average Coefficient	P value	Corrected P-Value
ber_mean	-0.043263	0.6954	0.777212
rms_mean	-0.032477	0.7592	0.801378
rms_var	-0.112743	0.2722	0.517180
zcr_mean	-0.235890	0.0462	0.219450
zcr_var	0.385616	0.0002	0.003800
tempo_mean	0.234341	0.0224	0.141867
tempo_var	0.098482	0.3416	0.588683
ban_mean	-0.143798	0.2376	0.509200
ban_var	0.095389	0.3718	0.588683
mean colorfulness	-0.040997	0.6832	0.777212
variance colorfulness	-0.142389	0.1792	0.509200
mean saturation	-0.130078	0.2396	0.509200
variance saturation	0.045518	0.6598	0.777212
mean luminosity	0.000503	0.9958	0.995800
variance luminosity	-0.133004	0.2228	0.509200
faces ratio	0.042038	0.6892	0.777212
Objects human area	-0.130870	0.2412	0.509200
Quality	0.051902	0.6100	0.777212
Scene frequency	0.358888	0.0004	0.003800

Note: Coefficients (β) represent average feature weights averaged across all N=100 Leave-One-Out (LOO) cross-validation iterations using standardized features. p-values were computed across iterations, and p-value adjustments were calculated using Benjamini–Hochberg False Discovery Rate (FDR) correction (α=.05) across all 19 combined features (10 visual, 9 auditory).

A12. Ridge Regression Results for Combined Model Predicting Liking

Reports average feature weights, raw p-values, and FDR-corrected p-values for the joint visual and auditory linear ridge regression predicting Liking ratings.

Table A9. Out-of-Sample Feature Coefficients for Combined Model (Liking)

Feature	Average Coefficient	P value	Corrected P-Value
ber_mean	-0.092802	0.3924	0.532543
rms_mean	0.104901	0.3126	0.494950
rms_var	0.028812	0.7748	0.865953
zcr_mean	-0.223261	0.0630	0.239400
zcr_var	0.287511	0.0084	0.055733
tempo_mean	0.006082	0.9556	0.955600
tempo_var	-0.010943	0.9136	0.955600
ban_mean	-0.199620	0.0934	0.253514
ban_var	-0.130202	0.2354	0.445636
mean colorfulness	-0.035334	0.7260	0.862125
variance colorfulness	-0.230528	0.0296	0.140600
mean saturation	-0.278012	0.0088	0.055733
variance saturation	0.158155	0.1222	0.257978
mean luminosity	0.182813	0.0916	0.253514
variance luminosity	-0.103974	0.3420	0.499846
faces ratio	-0.120393	0.2580	0.445636
Objects human area	0.075535	0.5024	0.636373
Quality	-0.156574	0.1082	0.256975
Scene frequency	0.319948	0.0024	0.045600

Note: Coefficients (β) represent average feature weights averaged across all N=100 Leave-One-Out (LOO) cross-validation iterations using standardized features. p-values were computed across iterations, and p-value adjustments were calculated using Benjamini–Hochberg False Discovery Rate (FDR) correction (α=.05) across all 19 combined features (10 visual, 9 auditory).

A13. Visual and Auditory Feature Correlation Matrix

Figure A3 shows a heat map of the correlation matrix Low-Level Visual and Auditory Features.

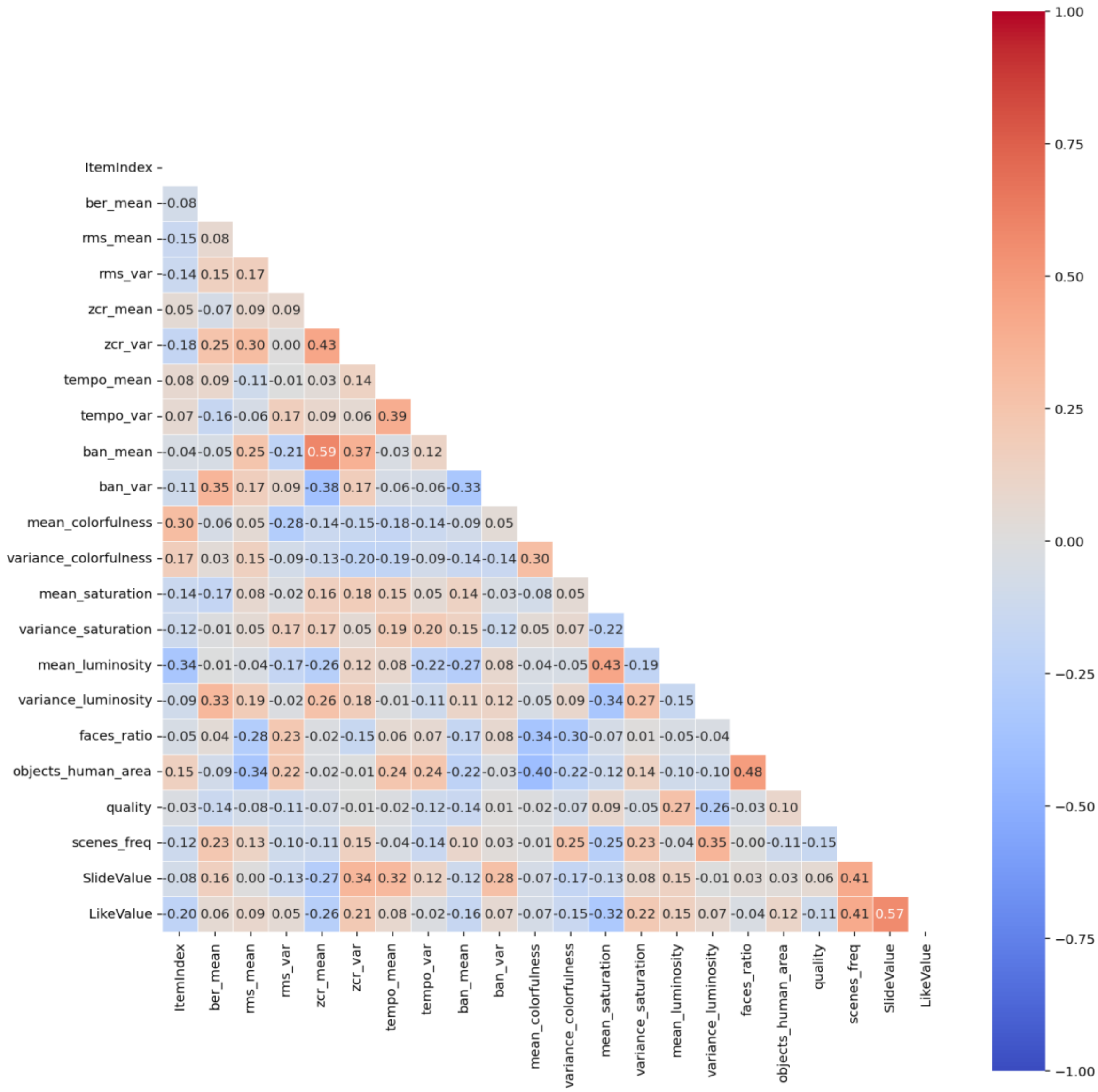

Figure A3. *Pearson correlation matrix of low-level visual and auditory features.*
Note. Heatmap displaying pairwise Pearson correlation coefficients across extracted low-level auditory features for the commercial stimuli (N = 100). Values range from -1.00 (strong negative correlation) to +1.00 (strong positive correlation), as indicated by the color spectrum (coolwarm). Numerical annotations within each cell represent rounded correlation coefficients.

A14. Main analysis without feature selection

We ran the main model using all features that provided a single measure per video, either directly or through aggregation across the entire video (48 features). As stated before, all the 14 features related to emotions had a lot of NaN values, so for this analysis we fill the NaNs with the mean of the column.

We predicted the average Liking and WTP ratings using ridge regression with leave-one-out (LOO) cross-validation. Feature selection substantially improved predictive

performance, increasing R^2 from 0.17 ($MSE = 0.787$, $MAE = 0.710$) with all features to $R^2 = 0.33$ ($MSE = 0.700$, $MAE = 0.664$) with selected features. In contrast, for WTP ratings, predictive accuracy was higher prior to feature selection ($R^2 = 0.21$, $MSE = 0.818$, $MAE = 0.639$) compared to post-selection ($R^2 = 0.17$, $MSE = 0.859$, $MAE = 0.740$)

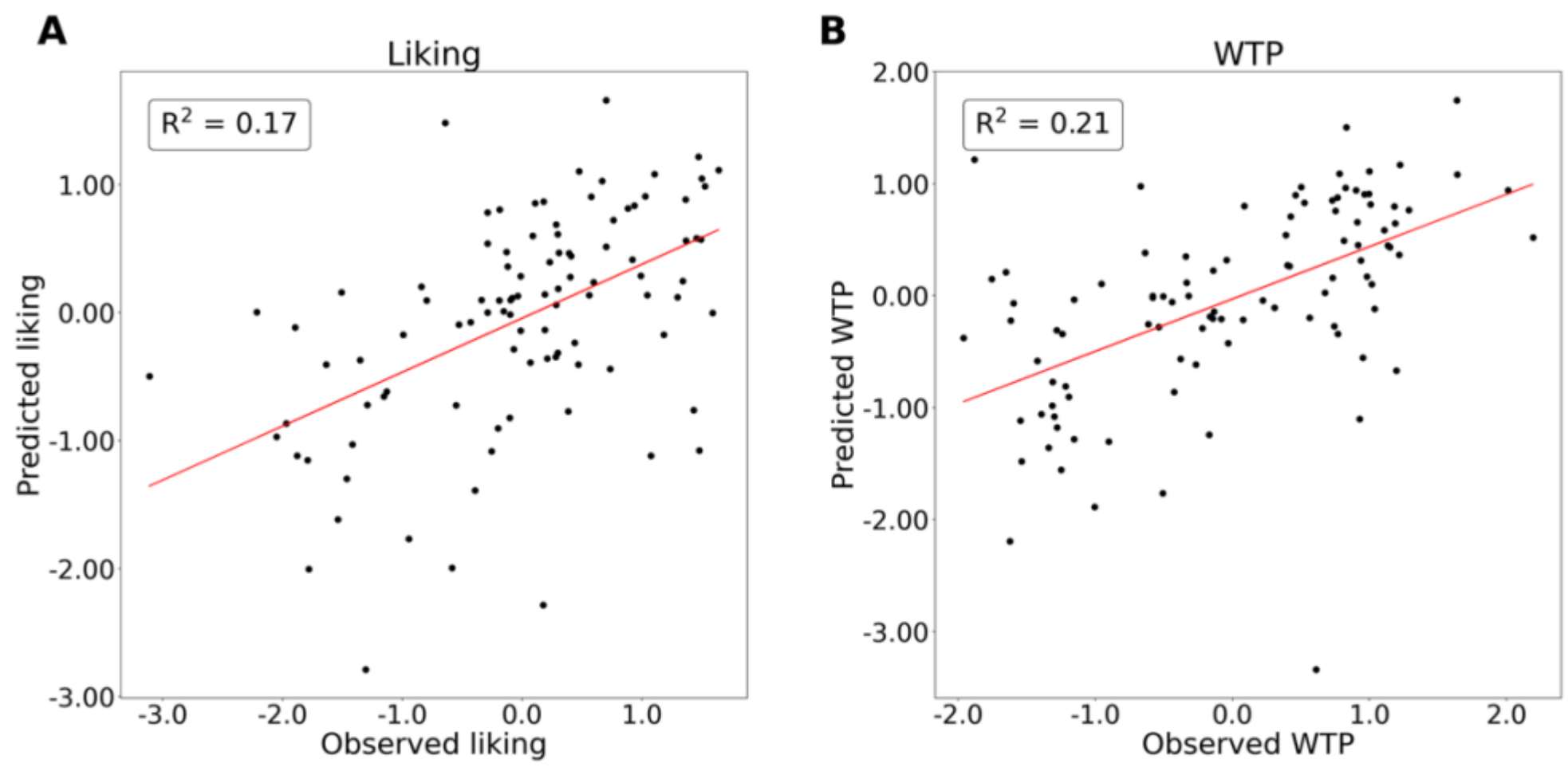

Fig A4. Prediction results for low-level visual features. The comparison of observed vs. predicted values for Liking (A) and WTP (B). Each dot represents the average value for a commercial across participants. The red line represents the best-fitted line. R^2 represents the average goodness-of-fit across all LOO iterations. Note, values are normalized.

We then averaged the beta coefficients across LOO iterations and tested each against a null distribution from 5,000 permutations with shuffled labels. Importantly, both before and after FDR correction, no individual feature was significant.

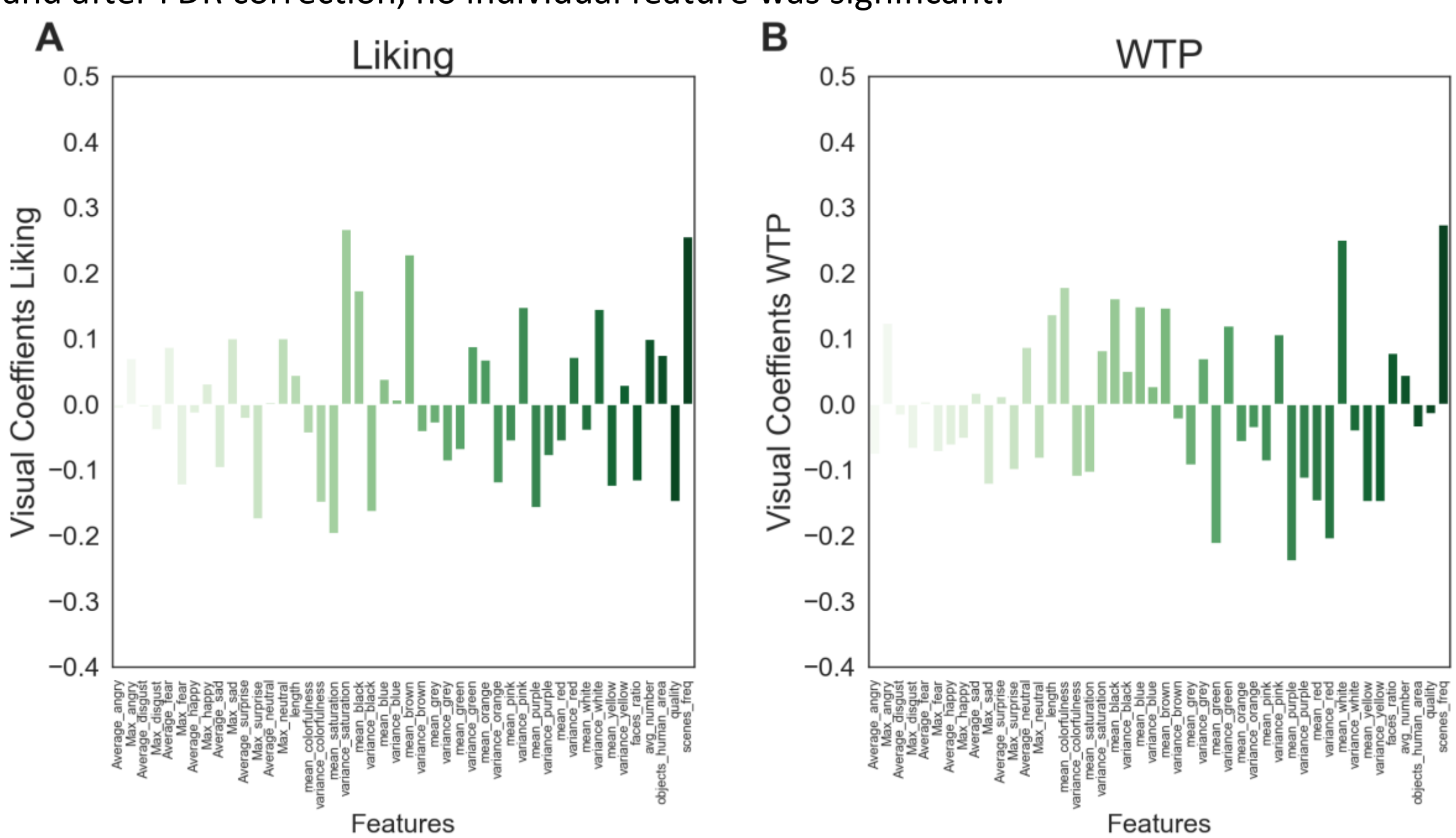

Fig A5. Low-level visual influential features. The most influential low-level visual features (x-axis) on subjective rankings of commercials – *Liking* (A) and WTP (B). The y-axis represents the values of the average coefficients from the LOO regressions. *** p<0.001, ** p<0.01, * p<0.05, all FDR corrected.

A15. Kernel Counts for the SVR Models

Table A10 reports the frequency of kernel selection across Support Vector Regression (SVR) models for each feature set and prediction target

Table A10. Kernel Selection Frequency

Feature Set	Target	rbf	linear	poly	sigmoid
**Visual**	Liking	942	40	17	1
	WTP	998	0	2	0
**Audio**	Liking	978	10	10	2
	WTP	997	1	1	0
**Audio-Visual**	Liking	969	31	0	0
	WTP	998	0	2	0

A16. Classification Task

Binary Classification Analysis

We used a binary classification model to get a discrete and quantitative sense of our ability to predict *Liking* and WTP of commercials in terms of prediction accuracies. Towards this aim, we used a SVM classifier. We divided separately the WTP or *Liking* ratings separately into high and low values using a median split and calculated the success of the SVM model to classify if a commercial was ranked high or low based on the low-level features as inputs to the model. As we did in the regression models, we trained two models (for WTP and *Liking*) using low-level visual features only, two models using low-level auditory features only, and two models using the combination of both features. Last, we compared each of the models to a model with shuffled subjective rankings. For robustness, we repeated these steps but using a logistic regression as the predictive model instead of the SVM model.

Overall Accuracy Results

As can be seen, both the SVM (Fig 4A) and the logistic regression (Fig 4B) models achieved relatively high prediction accuracies ranging between 61%-80%. Like in our previous models, the accuracy was higher for WTP than for *Liking*, models using visual features only were better than models using auditory features only and using both visual and auditory features resulted in the highest prediction accuracies. Importantly, all models were significantly better than the random models using the shuffled labels (all P<0.001).

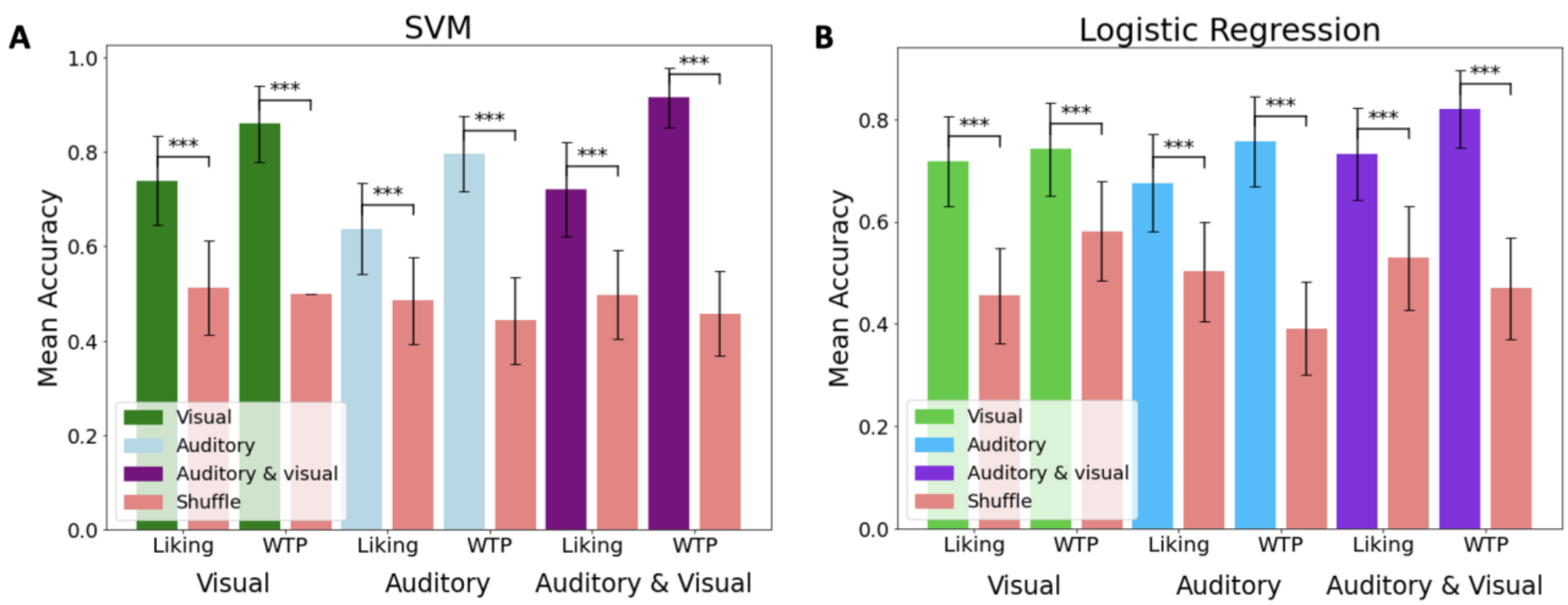

Figure A6. The mean accuracy scores of all the SVM and logistic regression models for the original and shuffled data.

A17. Performance Metrics of the SVM and Logistic Regression Models

Metrics of the SVM classifier models

In Table A11 (*Liking*) and table A12 (WTP), we report for each SVM and logistic regression model the accuracy, sensitivity, specificity, precision, and negative predictive value (NPV) of the test set.

Table A15. Performance metrics for predicting Liking

Type of Features	Model	*Mean values of the models for predicting Liking*				
		Accuracy	Sensitivity	Specificity	Precision	NPV
**Visual**	SVM	0.76	0.77	0.77	0.76	0.75
	Logistic	0.76	0.77	0.77	0.76	0.76
**Auditory**	SVM	0.64	0.64	0.69	0.70	0.60
	Logistic	0.65	0.65	0.65	0.64	0.65
**Visual & Auditory**	SVM	0.74	0.75	0.75	0.73	0.74
	Logistic	0.75	0.76	0.76	0.75	0.75

As can be seen in Table A11, for *Liking* values, the prediction success for both types of features (visual and auditory) in both model types (SVM and logistic regression) is high. However, they are higher when using visual features compared to auditory features. Moreover, there is no improvement when adding the auditory features to the visual features, suggesting that for binary classifications using these models, the visual features are contributing more to the prediction of *Liking* values.

Table A12. Performance metrics for predicting WTP

	Model	*Mean values of the models for predicting WTP*

Type of Features		Accuracy	Sensitivity	Specificity	Precision	NPV
**Visual**	SVM	0.80	0.81	0.81	0.80	0.80
	Logistic	0.73	0.74	0.74	0.73	0.73
**Auditory**	SVM	0.76	0.76	0.78	0.77	0.74
	Logistic	0.76	0.75	0.79	0.80	0.71
**Visual & Auditory**	SVM	0.86	0.90	0.84	0.82	0.90
	Logistic	0.80	0.80	0.82	0.81	0.79

As can be seen in Table A12, the overall prediction success of both models for WTP values is higher than for *Liking* values. In addition, for the *Liking* values, using visual features results in higher prediction success than when using the auditory features. However, here the combination of both visual and auditory features results in the highest prediction success values. These results suggest that low-level visual features are more influential for predicting WTP values compared to low-level auditory features, but that both features are related to WTP values of the products advertised in the commercials.
